# Combined unsupervised-supervised machine learning for phenotyping complex diseases with its application to obstructive sleep apnea

**DOI:** 10.1038/s41598-021-84003-4

**Published:** 2021-02-24

**Authors:** Eun-Yeol Ma, Jeong-Whun Kim, Youngmin Lee, Sung-Woo Cho, Heeyoung Kim, Jae Kyoung Kim

**Affiliations:** 1 Department of Industrial and Systems Engineering, Korea Advanced Institute of Science and Technology, Daejeon, Republic of Korea; 2 Department of Otorhinolaryngology, Seoul National University Bundang Hospital, Seoul National University College of Medicine, Seongnam, Republic of Korea; 3 Department of Mathematical Sciences, Korea Advanced Institute of Science and Technology, Daejeon, Republic of Korea

**Keywords:** Machine learning, Diseases, Risk factors

## Abstract

Unsupervised clustering models have been widely used for multimetric phenotyping of complex and heterogeneous diseases such as diabetes and obstructive sleep apnea (OSA) to more precisely characterize the disease beyond simplistic conventional diagnosis standards. However, the number of clusters and key phenotypic features have been subjectively selected, reducing the reliability of the phenotyping results. Here, to minimize such subjective decisions for highly confident phenotyping, we develop a multimetric phenotyping framework by combining supervised and unsupervised machine learning. This clusters 2277 OSA patients to six phenotypes based on their multidimensional polysomnography (PSG) data. Importantly, these new phenotypes show statistically different comorbidity development for OSA-related cardio-neuro-metabolic diseases, unlike the conventional single-metric apnea–hypopnea index-based phenotypes. Furthermore, the key features of highly comorbid phenotypes were identified through supervised learning rather than subjective choice. These results can also be used to automatically phenotype new patients and predict their comorbidity risks solely based on their PSG data. The phenotyping framework based on the combination of unsupervised and supervised machine learning methods can also be applied to other complex, heterogeneous diseases for phenotyping patients and identifying important features for high-risk phenotypes.

## Introduction

Obstructive sleep apnea (OSA) is one of the most common sleep disorders^1^ and is a risk factor of various diseases, including cardiovascular, neurovascular, and metabolic diseases^2–7^. The standard test for diagnosing OSA^8^ is based on the polysomnography (PSG)^9^, which records various parameters such as sleep architecture, respiratory events, oxygen desaturation, and limb movements during sleep. However, only the respiratory events are used for the conventional diagnosis of OSA. In particular, only the apnea–hypopnea index (AHI), which is the number of apneas (temporary cessation of breathing) and hypopneas (partial blockage of the airway) per hour of sleep, is used. This is also used to classify patients into phenotypes such as mild, moderate, and severe OSA (Supplementary Table S1). The prognosis of associated diseases is also made primarily based on the AHI^10^.

The AHI alone is likely an over-simplistic index to explain the heterogeneity and complexity of the disease. For instance, sole AHI fails to discriminate a patient’s comorbidity outcomes within the same AHI severity^10–13^. This indicates the need for more comprehensive phenotyping of OSA beyond just the AHI (Supplementary Table S1)^14^. Recently, more comprehensive OSA phenotypes have been identified through clustering PSG data (e.g. graph-based clustering^15^ and K-means clustering^16,17^), which is well-suited for OSA phenotyping because PSG data is multidimensional data containing various information regarding the patient’s sleep generated during their initial visit for diagnosis. For example, the K-means algorithm was used to cluster OSA patients into seven phenotypes based on their PSG^17^. This identified a cluster of patients with high periodic limb movements (PLM), which is an important PSG feature. Interestingly, this cluster had significantly higher risks of cardiovascular diseases among clusters with a mild degree of AHI. This indicates that the AHI alone is not enough to explain the comorbidity developments in OSA patients, and highlights the need for OSA phenotyping based on all PSG data.

To analyze multidimensional clinical data such as PSG, cluster analysis has been widely used. This has uncovered new phenotypes of complex and heterogeneous diseases including not only OSA^15–18^ but also other diseases such as asthma^19–22^, chronic obstructive pulmonary disease^23,24^, chronic heart failure^25^, sepsis^26^, Parkinson’s disease^27^, and diabetes^28,29^. However, the clustering algorithms used in these studies require the number of clusters to be manually determined either before (e.g. K-means) or after (e.g. hierarchical clustering) clustering. As a result, for example, a variable number of OSA patient clusters (3 to 7)^15–18^ and asthma patient clusters (3 to 6) were chosen in previous studies^19–22^. Choosing the number of clusters is often made based on clinical intuition^15,19,21,22^, which can be subjective. Although less subjective model selection criteria (e.g. silhouette width, likelihood ratio, Bayesian information criterion) have been also used^16,17,20^, the optimal number of clusters can change depending on the choice of the selection criteria^30–32^. Moreover, these model selection criteria often require high computational costs because repeated experiments are needed when searching for the optimal number of clusters^30–32^. They also become unreliable when data are noisy and complex. The choice of the number of clusters is particularly important in phenotyping as it can dramatically change the phenotyping results^15,25,33^. For example, as a larger number of clusters is chosen, a more precise phenotype may be obtained, but each discovered cluster may not have enough patients to accurately describe the pathophysiology of the phenotype^25^.

The next critical step after clustering patients is identifying the key cluster features leading to the outcomes of interest (e.g., comorbidity, survival, or hospitalization) for prognosis and prevention^14,34^. This is not straightforward using methods solely based on cluster analysis because relationships between input features and outcomes are not estimated during the training process of clustering. Thus, the interpretation of the associated comorbidity risks of the clusters was limited to subjective inspections in previous studies for various diseases including OSA, asthma, chronic obstructive pulmonary disease, chronic heart failure, and diabetes^15,17,22,24,25,28^.

Here, to circumvent such limitations of cluster-based phenotyping, we developed a multimetric phenotyping framework based on a combination of unsupervised and supervised machine learning algorithms. Specifically, in order to cluster PSG data and discover new phenotypes using only the readily available multidimensional PSG data without predetermining the number of clusters, we used Bayesian nonparametric clustering, which has been successful at clustering patient data of various diseases^29,35,36^. Furthermore, instead of subjectively and manually selecting cluster features, we used survival prediction models to identify highly-confident cluster features of the comorbid clusters. This reveals the complex aspects of OSA beyond the single AHI metric and the importance of using all PSG data to diagnose OSA patients for the better prognosis of associated comorbidities. We also develop a computational package that can phenotype new OSA patients solely based on their PSG data with the trained models from our framework (https://github.com/Mathbiomed/OSA-phenotyping/). Our work highlights the need for the combined use of unsupervised and supervised models for clinical phenotyping.

## Results

### Study pipeline

**Figure 1:**
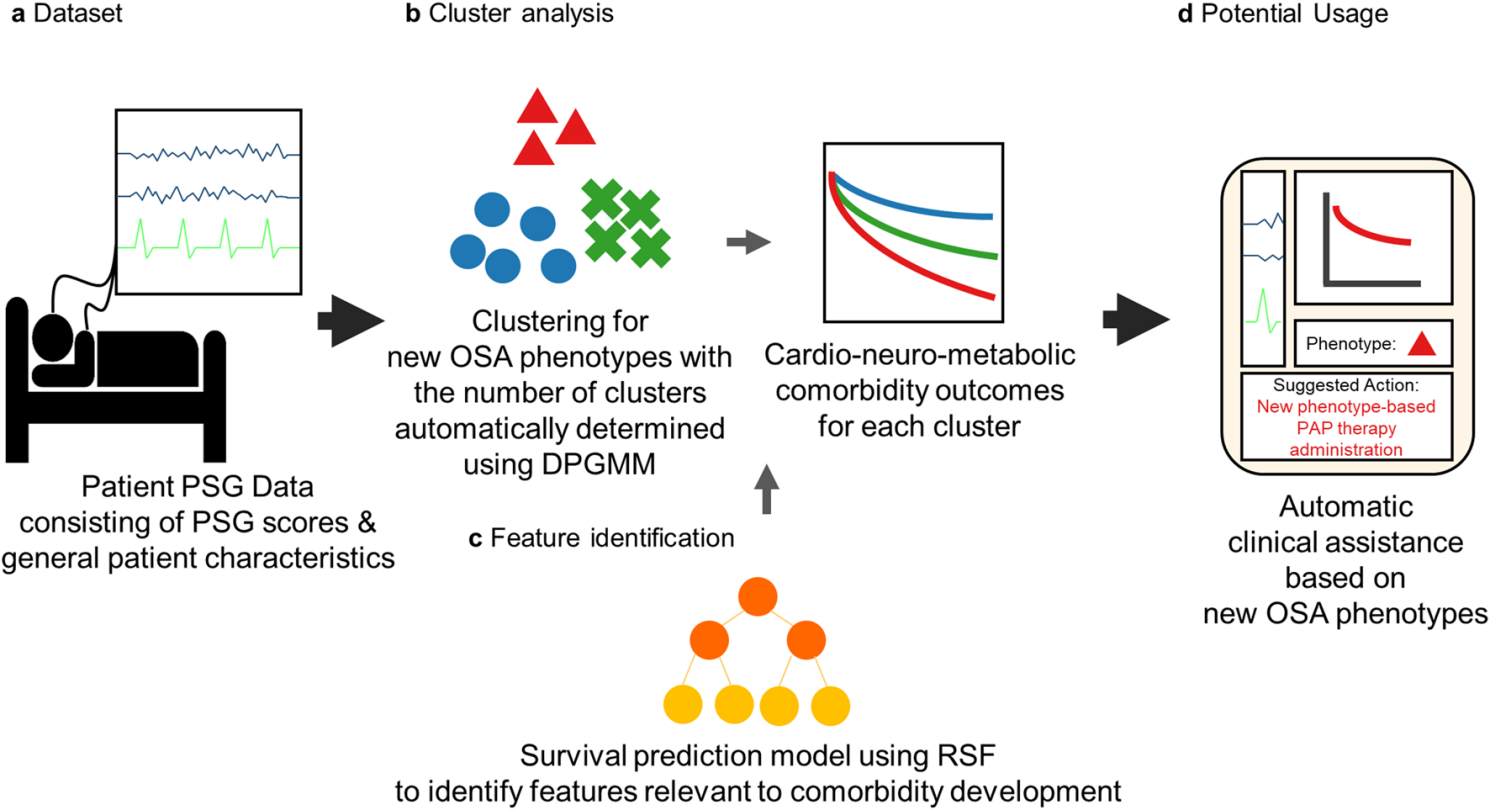
Overall study pipeline. (**a**) The dataset includes PSG scores of the patient cohort. (**b**) OSA patients are clustered based on the PSG data using the DPGMM, which determines the number of clusters automatically during the clustering procedure, and each cluster had different cardio-neuro-metabolic comorbidity outcomes. (**c**) Key cluster features were identified based on features with high importance in predicting comorbidity risks according to the RSF. (**d**) Results can be integrated into the PSG system for automatic clinical assistance, which can improve the diagnosis and treatment of OSA and the associated cardio-neuro-metabolic diseases. PAP, positive airway pressure.

The PSG data of 2277 patients from a tertiary hospital in Korea was used in the study (Fig. 1a). Throughout the paper, we refer to the whole data including both the PSG scores and the general patient characteristics as PSG data. We used the Dirichlet process Gaussian mixture model (DPGMM) for the unsupervised clustering-based phenotyping of OSA patients (Fig. 1b) because the optimal number of clusters (phenotypes) is determined during the clustering procedure instead of being predetermined (see “Methods” for details). In order to identify the key features of the highly comorbid clusters, we also developed a supervised survival prediction model using the random survival forest (RSF) (Fig. 1c) and found features highly related to comorbidity prevalence. The phenotyping framework may be integrated into the PSG system for automatic clinical assistance (Fig. 1d).

### DPGMM identified six OSA phenotypes with distinguishing PSG features

**Figure 2:**
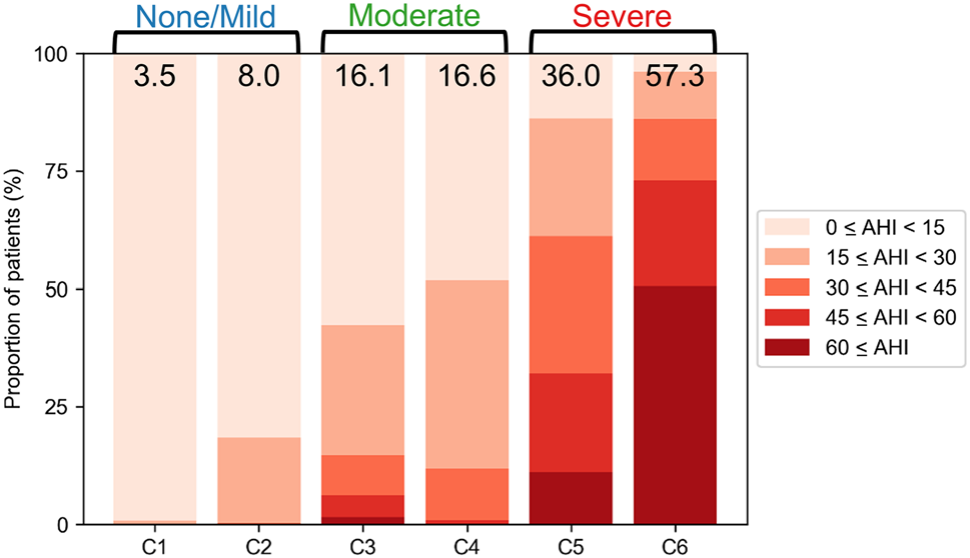
The distribution of AHI within DPGMM-created clusters (n = 2277). The mean AHI of each cluster is written on the top of each bar.

Due to the high dimensionality of the PSG data and the likelihood that many of the features are correlated (and thus to a certain extent redundant), we applied principal component analysis, which reduced the original 43 features to eight principal components explaining up to 70% of the total data variance (see “Methods” section for details). We clustered the 2277 OSA patients based on these eight extracted features by using K-means clustering, which is one of the most commonly used clustering algorithms for clinical phenotyping^16,17,19,24,28^. However, the optimal number of clusters dramatically changes depending on the model selection criteria (Supplementary Table S2), which makes it difficult to determine the appropriate number of clusters based on the criteria: the silhouette width criterion suggests few clusters while the Bayesian information criterion suggests many. In addition, the silhouette score monotonically decreases with an increasing number of clusters, indicating highly overlapping data. This makes K-means clustering unsuitable because it can only generate spherical and non-overlapping clusters and hence cannot identify the true data structure^37^. To avoid these issues, we used the DPGMM, which does not require the predetermination of the number of clusters and discovers various ellipsoidal clusters on overlapping data^37^. Using the DPGMM, the optimal number of clusters was identified as six, which was the number of clusters discovered for the majority of repeated experiments (Table 1, Supplementary Table S3, and Supplementary Table S4). The discovered six clusters were labeled in order of increasing mean AHI: the mean AHI of C1 (n = 254, mean (± SD) AHI = 3.5 ± 4.0) and C2 (n = 304, mean AHI = 8.0 ± 7.1) fell in the no/mild OSA region, the mean AHI = C3 (n = 290, mean AHI = 16.1 ± 14.9) and C4 (n = 351, mean AHI = 16.6 ± 10.5) fell in the moderate region, and the mean AHI of C5 (n = 585, mean AHI = 36.0 ± 18.8) and C6 (n = 360, mean AHI = 57.3 ± 23.5) fell in the severe region. The proportion of patients with moderate or severe OSA according to the conventional AHI standards increased accordingly as well (Fig. 2). Despite each pair of clusters (e.g. C1 and C2) having similar mean AHI, the clusters had distinct PSG features (Supplementary Table S5).

**Table 1:** Feature means (SD) of DPGMM-created clusters (n = 2277).

	C1	C2	C3	C4	C5	C6	p-value
n	263	327	326	365	608	388	
Age	36.4 (16.5)	54.3 (16.3)	60.1 (12.1)	45.8 (13.7)	47.5 (11.5)	52.8 (13.8)	< 0.001
Body mass index (BMI)	23.3 (3.3)	23.7 (2.9)	25.3 (3.5)	24.6 (2.7)	27.8 (3.2)	27.3 (4.4)	< 0.001
Neck circumference	34.9 (3.3)	35.0 (3.1)	36.9 (3.4)	37.1 (2.7)	39.4 (2.7)	39.3 (3.4)	< 0.001
Waist-hip ratio	0.9 (0.1)	0.9 (0.1)	0.9 (0.1)	0.9 (0.0)	1.0 (0.0)	0.9 (0.0)	< 0.001
Pittsburgh sleep quality index (PSQI)	7.9 (4.3)	9.8 (4.9)	8.3 (4.4)	7.1 (3.8)	7.1 (3.6)	8.0 (4.1)	< 0.001
Epworth sleepiness scale (ESS)	10.0 (5.3)	7.6 (5.2)	8.1 (5.1)	9.6 (5.0)	9.9 (5.0)	9.9 (5.3)	< 0.001
Sleep latency (min)	16.1 (20.6)	22.5 (25.8)	23.4 (30.8)	10.8 (11.3)	11.8 (13.4)	25.0 (34.5)	< 0.001
Sleep period time (min)	445.9 (42.4)	444.7 (47.6)	438.3 (52.9)	446.6 (30.1)	448.7 (37.3)	422.6 (72.9)	< 0.001
Wake time after sleep onset (WASO) (min)	32.8 (21.0)	93.6 (42.4)	85.0 (50.0)	52.0 (28.8)	62.8 (38.2)	94.9 (60.6)	< 0.001
Total sleep time (min)	414.1 (48.0)	356.7 (52.2)	358.2 (58.3)	396.1 (36.7)	387.5 (45.1)	334.2 (79.0)	< 0.001
Sleep efficiency (%)	89.4 (6.7)	75.5 (10.0)	77.0 (11.5)	86.5 (6.9)	84.0 (8.6)	73.9 (14.9)	< 0.001
REM latency (min)	109.8 (59.2)	138.7 (78.3)	139.7 (88.8)	110.7 (59.8)	126.8 (69.7)	145.8 (88.4)	< 0.001
Proportion of N1 sleep (N1) (%)	5.9 (3.2)	9.3 (4.6)	10.9 (6.1)	9.6 (4.9)	14.2 (7.4)	19.9 (11.0)	< 0.001
Proportion of N2 sleep (N2) (%)	54.4 (10.1)	46.2 (10.2)	49.6 (11.3)	51.2 (8.4)	48.8 (10.8)	43.8 (14.0)	< 0.001
Proportion of N3 sleep (N3) (%)	12.9 (9.3)	10.0 (7.8)	6.7 (7.5)	9.9 (7.1)	7.3 (5.9)	3.9 (5.7)	< 0.001
Proportion of REM sleep (REM) (%)	19.9 (7.2)	14.9 (6.4)	14.8 (6.4)	18.0 (6.1)	16.1 (5.8)	11.6 (6.2)	< 0.001
Apnea–hypopnea index (AHI) (/h)	3.5 (4.0)	8.0 (7.1)	16.1 (14.9)	16.6 (10.5)	36.0 (18.8)	57.3 (23.5)	< 0.001
Apnea index (/h)	1.0 (1.5)	2.8 (3.6)	8.0 (10.2)	8.4 (7.8)	20.9 (16.2)	45.4 (24.4)	< 0.001
Obstructive apnea (/h)	0.7 (1.5)	2.4 (3.3)	6.8 (9.1)	7.6 (7.4)	18.2 (7.4)	37.1 (23.3)	< 0.001
Central apnea (/h)	0.2 (0.4)	0.3 (0.6)	0.4 (1.1)	0.4 (0.7)	0.9 (0.7)	1.8 (4.5)	< 0.001
Mixed apnea (/h)	0.0 (0.1)	0.2 (0.4)	0.8 (2.5)	0.5 (1.1)	2.0 (3.9)	6.5 (10.1)	< 0.001
Hypopnea index (/h)	2.5 (3.1)	5.2 (5.3)	8.1 (7.6)	8.2 (6.4)	15.1 (10.0)	11.9 (11.7)	< 0.001
REM AHI (/h)	5.9 (8.0)	10.5 (13.3)	18.3 (19.2)	20.7 (16.0)	38.1 (22.1)	46.2 (25.3)	< 0.001
NREM AHI (/h)	2.8 (3.8)	7.5 (7.4)	15.5 (15.4)	15.3 (11.2)	35.5 (20.2)	58.9 (24.3)	< 0.001
Supine AHI (/h)	5.1 (6.2)	13.4 (13.3)	25.9 (24.3)	24.7 (16.9)	51.3 (25.0)	64.9 (23.4)	< 0.001
Lateral AHI (/h)	1.3 (2.5)	2.7 (5.5)	10.6 (19.6)	6.2 (10.4)	25.0 (32.0)	53.0 (57.6)	< 0.001
Longest apnea duration (s)	19.5 (15.4)	26.6 (17.1)	35.9 (21.7)	44.8 (24.4)	46.2 (19.3)	66.0 (27.9)	< 0.001
Mean apnea duration (s)	13.6 (8.8)	16.7 (8.8)	19.3 (8.0)	23.3 (9.0)	22.1 (5.9)	29.4 (9.3)	< 0.001
Mean hypopnea duration (s)	22.0 (12.7)	24.9 (9.7)	26.1 (7.2)	29.1 (8.2)	25.5 (5.0)	27.3 (8.6)	< 0.001
Mean total AH duration (s)	20.7 (10.2)	23.5 (8.0)	24.9 (5.9)	27.6 (7.3)	24.5 (4.6)	30.4 (7.9)	< 0.001
Average O_2_ saturation (%)	96.8 (1.2)	96.1 (1.4)	95.4 (1.6)	96.1 (1.3)	94.7 (1.5)	92.7 (3.1)	< 0.001
Lowest O_2_ saturation (%)	90.2 (4.0)	88.6 (4.3)	85.5 (6.2)	84.4 (5.7)	79.5 (6.8)	74.4 (9.6)	< 0.001
Proportion of sleep spent under 90% O_2_ saturation (T90) (%)	0.2 (0.7)	0.4 (1.0)	2.2 (5.0)	1.6 (3.3)	6.0 (7.4)	20.2 (19.2)	< 0.001
Oxygen desaturation index (ODI) (/h)	2.1 (2.8)	5.0 (5.4)	12.0 (13.1)	11.4 (8.6)	29.9 (18.1)	51.3 (23.7)	< 0.001
Snoring time (%)	9.6 (11.2)	8.8 (9.7)	24.4 (22.3)	29.4 (19.2)	39.9 (19.8)	19.6 (13.0)	< 0.001
Number of snoring episodes	35.3 (38.0)	37.1 (41.1)	79.4 (65.3)	96.8 (55.4)	167.3 (86.1)	189.8 (137.8)	< 0.001
Average snoring duration (min)	0.9 (0.8)	0.7 (0.6)	1.1 (0.9)	1.4 (1.3)	1.1 (0.9)	0.4 (0.3)	< 0.001
Longest snoring duration (min)	6.5 (7.2)	5.3 (5.6)	12.5 (12.2)	17.5 (13.3)	19.7 (14.6)	4.9 (6.1)	< 0.001
Limb movement (/h)	5.9 (4.9)	10.9 (9.9)	56.8 (31.5)	7.2 (6.6)	10.8 (9.9)	21.2 (18.8)	< 0.001
Periodic limb movement (PLM) (/h)	0.9 (2.5)	3.9 (6.6)	39.7 (27.4)	0.5 (1.3)	0.9 (2.7)	1.4 (4.6)	< 0.001
Respiratory arousal (/h)	2.1 (2.9)	5.3 (5.2)	11.2 (11.8)	11.8 (8.7)	25.7 (16.1)	48.1 (22.1)	< 0.001
PLM arousal (/h)	0.2 (0.7)	0.7 (1.5)	6.9 (9.3)	0.1 (0.2)	0.1 (0.4)	0.1 (0.5)	< 0.001
Spontaneous arousal (/h)	6.1 (3.6)	6.2 (3.9)	4.1 (5.7)	5.1 (3.2)	3.5 (2.5)	2.0 (2.7)	< 0.001

Clusters were labeled in the order of increasing mean AHI. Omnibus analysis of variance was conducted for statistical comparisons.

### Clusters with low AHI but high comorbidity prevalence were identified

**Figure 3:**
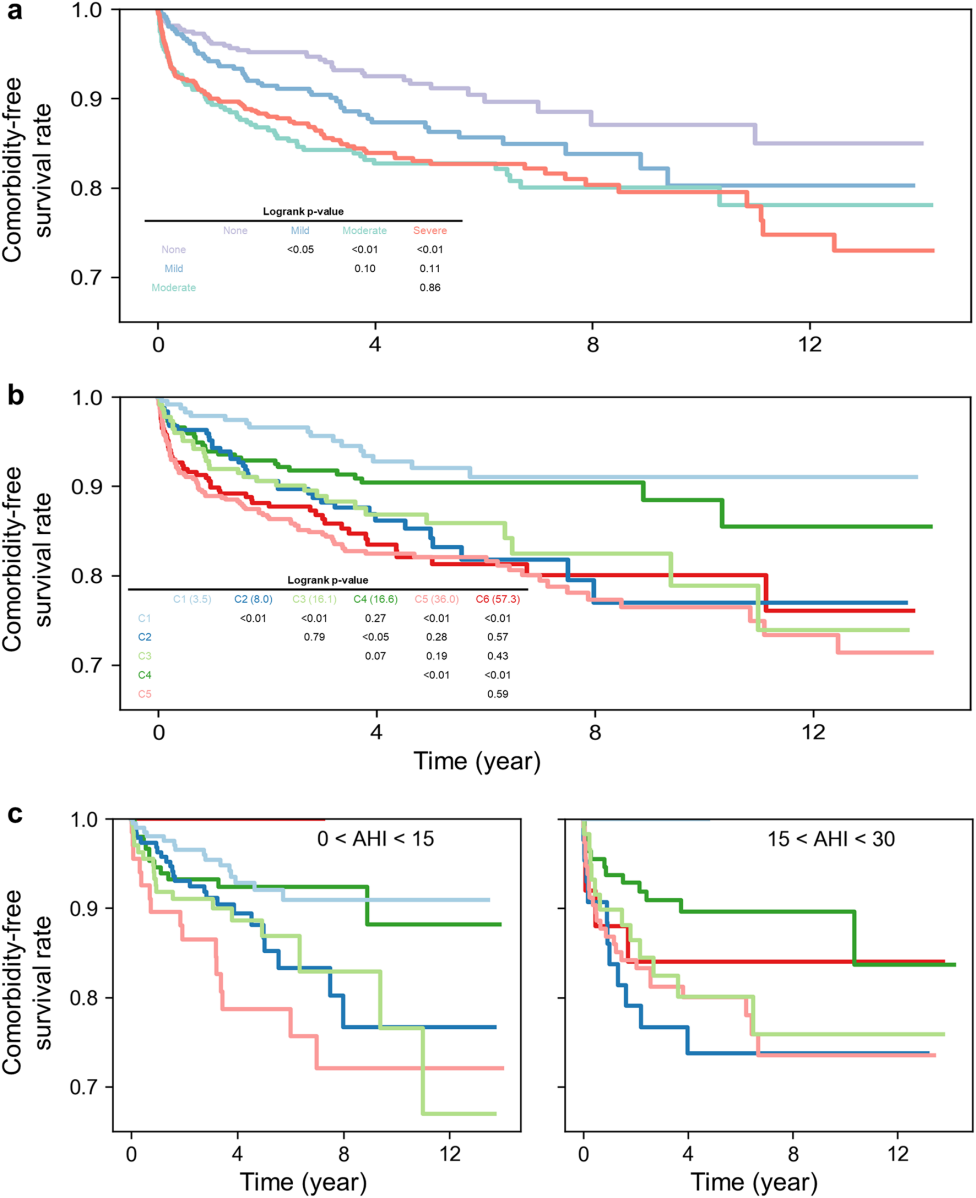
Kaplan–Meier curves of the cardio-neuro-metabolic comorbidity outcomes of the patient cohort (n = 1754). Patients diagnosed with the comorbidities within a year prior to the PSG test were excluded. The average follow-up for our data was 5.0 ± 3.4 years. (**a**) The comorbidity-free survival curves of the conventional AHI-based phenotypes. The pairwise logrank p-values between the mild, moderate, and severe groups were not statistically significant (Mild vs moderate p-value = 0.10, mild vs severe p-value = 0.11, moderate vs severe p-value = 0.86). Multivariate logrank p-value < 0.001. (**b**) The comorbidity-free survival curves of the DPGMM-created clusters. C1 and C4 had a high comorbidity-free survival rate, while C2, C3, C5, and C6 had a low comorbidity-free survival rate (pairwise logrank test C1 vs. C2/C3/C5/C6 p-value < 0.001, C4 vs. C2 p-value = 0.04, C4 vs. C3 p-value = 0.07, C4 vs. C5/C6 p-value < 0.001). Multivariate logrank p-value < 0.001. (**c**) The comorbidity-free survival curves of the DPGMM-created clusters were constructed with only the patients who fall within the specified AHI range. The survival rates of C2 and C3 were still lower than that of C1 and C4. Multivariate logrank p-value < 0.01.

The prevalence of OSA comorbidities such as cardiovascular, neurovascular, and metabolic diseases is known to increase with increasing AHI in general^5,6^. Consistent with this, the patients in our cohort diagnosed with OSA using AHI (AHI ≥ 5) had a significantly higher prevalence of cardio-neuro-metabolic diseases than the patients who were not diagnosed with OSA (0 ≤ AHI < 5) (pairwise logrank p-values none vs mild p-value = 0.04, none vs moderate p-value < 0.001, none vs severe p-value < 0.001) (Fig. 3). The decrease in survival rate as the AHI severity increased was also observable (Fig. 3a), but the differences between the groups were limited and not statistically significant (pairwise logrank p-values mild vs moderate p-value = 0.10, mild vs severe p-value = 0.11, moderate vs severe p-value = 0.86).

We next investigated whether the comorbidity outcomes were different among the patient clusters identified based on the expanded PSG data using our approach (Fig. 3b). Overall, the average follow-up time of each cluster is similar (Supplementary Table S6). C1 had the highest comorbidity-free survival rate, followed by C4. The other four clusters—C2, C3, C5, and C6—had similarly low comorbidity-free survival rates (pairwise logrank p-value > 0.1 for all pairs), with C5 and C6 having a faster decrease in survival rates prior to 5 years of follow-up. Interestingly, although C1 and C2 had similar mean AHI, C2 displayed a much higher comorbidity prevalence than C1 (pairwise logrank p-value < 0.01). Likewise, despite similar mean AHI, C3 also displayed a higher comorbidity prevalence than C4 (pairwise logrank p-value = 0.07). Note that although the mean AHI of C2 and C3 were of the mild and moderate levels respectively, they were as comorbid as C5 and C6, whose mean AHI was at the severe level. Because the clusters consist of patients with diverse AHI levels (Fig. 2), we wondered whether it was the patients of C2 and C3 with high AHI levels that were the major source of the high comorbidity prevalence of these clusters. To investigate this, we recalculated the Kaplan–Meier curves of the clusters with only the patients whose AHI belongs to the intervals 0 ≤ AHI < 15 and 15 ≤ AHI < 30 (Fig. 3c), as C1, C2, C3, and C4 mostly consist of patients with AHI within these intervals. Even when the patients with similar AHI levels were compared, the results were consistent with the comorbidity outcomes of the full cohort (Fig. 3b,c); the survival rates of C2 and C3 were still lower than that of C1 and C4. This indicates that it was not the individual patients with high AHI in each cluster who were comorbid, but that the patients constituting the cluster generally developed comorbidities. The results indicate that patients with similar AHI can have dramatically different comorbidities depending on the cluster they belong to, which is determined by their PSG characteristics. In turn, patients with different AHI can have similar comorbidities. This explains why the conventional phenotypes based solely on AHI lead to only marginally different survival curves (Fig. 3a).

For the patients whose smoking and drinking status were available, we also calculated the proportion of patients who smoke and drink in each cluster to investigate whether the difference in the comorbidity prevalence between clusters with similar AHI (i.e. C1 vs C2 and C3 vs C4) is due to the different proportion of patients who smoke and/or drink (Supplementary Table S7). The difference in the proportion of patients who smoke between C1 and C2 and C3 and C4 was not statistically significant. Furthermore, the difference in the proportion of patients who drink between C3 and C4 was also not statistically significant. Thus, it appears that the higher comorbidity prevalence of the clusters C2 and C3 compared to C1 and C4 was not due to the difference in smoking and/or drinking.

### The RSF identified key cluster features relevant to comorbidity development

Consistent with previous studies^15–17,38^, our cluster analysis based on PSG data identifies OSA as a highly heterogeneous disease, which cannot be categorized solely by AHI, and that incorporating more PSG features helps better distinguish the comorbidity outcomes of OSA patients (Fig. 3a,b). However, cluster analysis does not identify the specific PSG feature(s) explaining the cluster’s comorbidity outcomes. For instance, among the various sleep characteristics of C3 such as high age, high PLM, and long sleep latency (Table 1), we cannot determine the key features contributing to the prevalence of comorbidities.

**Figure 4:**
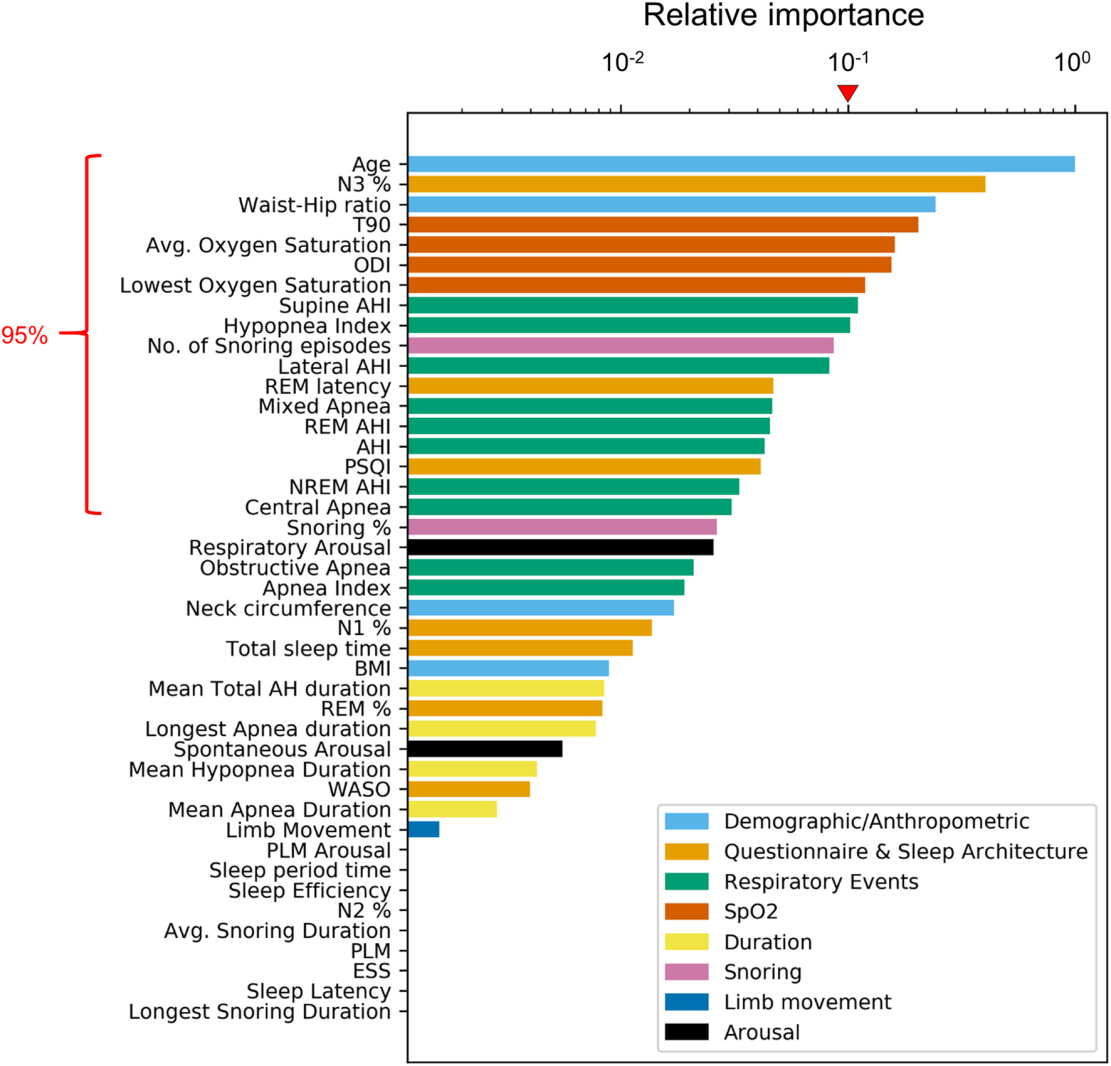
The relative importance of the PSG features for comorbidity risk prediction by the RSF (n = 1754). The absolute importance for each feature was calculated through the difference between the out-of-bag prediction accuracy of the model trained on true data and the model trained on randomly permuted data for the feature. The relative feature importance was calculated by dividing the absolute feature importance of each feature by that of the feature with the highest importance, age. The top 18 features (age → central apnea) accounted for 95% of the total importance. The red mark represents the relative importance of 0.1.

To overcome this limitation of clustering analysis, we additionally performed prediction analysis which utilizes labels in the training process and thus provides the relationship between the PSG data and comorbidity outcomes. Specifically, we performed survival prediction analysis on the full patient cohort by using the RSF: 43 PSG features (Table 1) were used as the input and the cardio-neuro-metabolic comorbidity outcomes were used as the label. The RSF provides the importance of each feature (Fig. 4) in predicting comorbidity risks (fivefold cross-validation concordance index = 0.65, integrated Brier score = 0.13), where features with greater importance can be considered more relevant to the comorbidity outcomes for our patient cohort.

**Figure 5:**
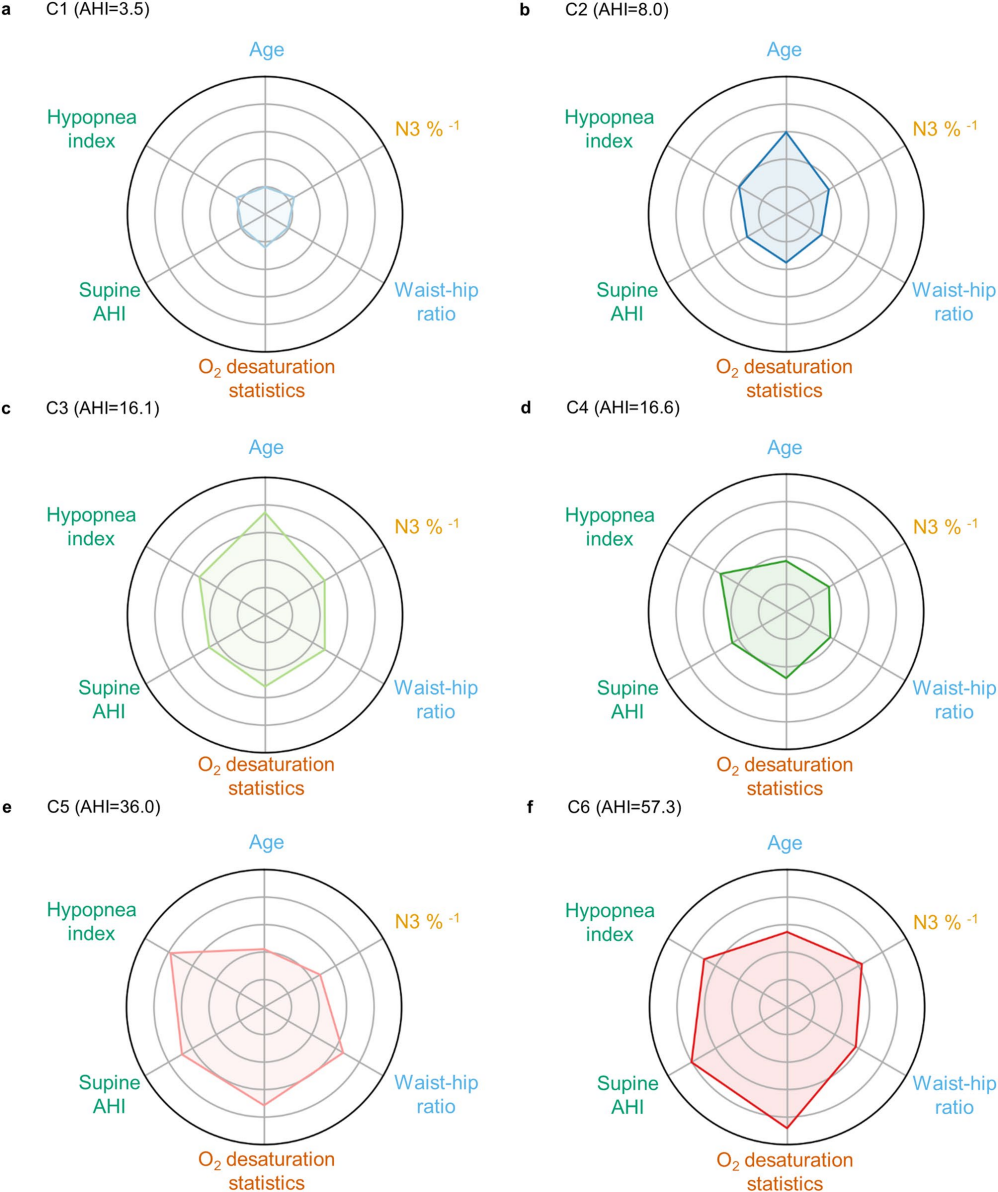
Radar plot of the DPGMM-created clusters with features found important by the RSF. Features with relative importance above 0.1 were chosen as the axes (Fig. 4). The percentile rank of the cluster means with respect to the whole patient data were plotted. Values were inversed for features that were “the-higher-the-better” so that all features were consistently the-lower-the-better. Oxygen desaturation statistics are shown through the mean of the percentiles of the four identified features (sleep time spent under 90% oxygen saturation, average oxygen saturation, oxygen desaturation event index, and lowest oxygen saturation) as these features represent similar clinical information. (**a**) Radar plot of C1 (mean AHI = 3.5), (**b**) radar plot of C2 (mean AHI = 8.0), (**c**) radar plot of C3 (mean AHI = 16.1), (**d**) radar plot of C4 (mean AHI = 16.6), (**e**) radar plot of C5 (mean AHI = 36.0), (**f**) radar plot of C6 (mean AHI = 57.3).

Among 43 PSG features, 18 features accounted for 95% of the total importance in predicting comorbidity outcomes (Fig. 4 and Supplementary Table S8). They included features regarding demographic and anthropometric characteristics (age, waist-hip ratio), sleep architecture and quality (the proportion of N3 sleep, REM latency, the Pittsburgh sleep quality index), oxygen desaturation (sleep time spent below 90% oxygen saturation, average oxygen saturation, oxygen desaturation event index, lowest oxygen saturation), respiratory events (supine AHI, hypopnea index, lateral AHI, mixed apnea, REM AHI, AHI, NREM AHI, central apnea), and snoring (number of snoring episodes). Age had the highest importance among all of the PSG features, followed by the proportion of N3 sleep and waist-to-hip ratio. All four features regarding oxygen desaturation came next. Supine AHI and hypopnea index followed the features regarding oxygen desaturation and were the features with the highest importance among respiratory events. Note that features regarding respiratory events, which are the conventional method of diagnosing OSA, were less important than expected. With the exception of the number of snoring episodes, features regarding snoring, respiratory event duration, limb movement, and arousal did not belong to the 18 features. In particular, features conventionally considered important phenotypic characteristics of OSA, such as body mass index, PLM, and respiratory event duration, which were often used to describe OSA clusters^17,18,39,40^, were of relatively less importance. Thus, in contrast to previous studies that have relied on inspection and subjective choice of features, we compared the clusters identified through the DPGMM based on the features with a relative importance of 0.1 or higher (Fig. 5).

#### Clusters with no or mild level of AHI: C1 and C2

C1 (mean AHI = 3.5 ± 4.0), which had the lowest comorbidity prevalence among all of the clusters (Fig. 3b), consisted of generally young patients with a low waist-to-hip ratio, satisfactory sleep quality, and low respiratory disturbance (Fig. 5a). On the other hand, C2 (8.0 ± 7.1), which had high comorbidity prevalence despite having a mild degree of AHI and a similar proportion of patients with no or mild OSA as C1 (Fig. 2), had worse scores for all 6 features (Fig. 5b). Specifically, in addition to having more respiratory events and higher oxygen desaturation, it had shorter N3 sleep, a higher age, and a higher waist-to-hip ratio than C1.

#### Clusters with a moderate level of AHI: C3 and C4

C3 (16.1 ± 12.1), which was a highly comorbid group (Fig. 3b), displayed characteristics similar to C2 but with poorer scores (Fig. 5c). Hence, along with a moderate degree of AHI, it had a relatively lower proportion of N3 sleep, a higher waist-to-hip ratio, and higher oxygen desaturation than C2. This cluster had the highest mean age among all the clusters identified. In contrast, C4 (16.6 ± 10.5), which had a low comorbidity prevalence, consisted of patients with a moderate level of respiratory events that were relatively younger and with a lower waist-to-hip ratio (Fig. 5d). The proportion of N3 sleep was also relatively high compared to C3. Although the differences were minimal, C4 had lower oxygen desaturation than C3 as well.

#### Clusters with a severe level of AHI: C5 and C6

C5 (36.0 ± 18.8) and C6 (57.3 ± 23.5) had very high respiratory disturbance and oxygen desaturation (Fig. 5e,f) and were clusters of high comorbidity prevalence (Fig. 3b). They also had short N3 sleep and a high waist-to-hip ratio. Although C6 was more extreme than C5 regarding most of the features identified as important by the RSF, C5 had a higher waist-to-hip ratio and hypopnea index than C6.

In summary, the features with high importance were able to appropriately describe the clusters and differentiate clusters with high and low comorbidity prevalence. The shape of the radar plots of the highly comorbid clusters (i.e. C2 and C3) were similar (Fig. 5b,c), and the size of the plots were larger than that of the clusters with low comorbidity prevalence with similar AHI (i.e. C1 and C4) (Fig. 5a–d). This indicates that patients displaying PSG characteristics such as high age, a low proportion of N3 sleep, high waist-hip-ratio, and high oxygen desaturation require closer monitoring.

Because age was the most important feature, and highly comorbid clusters displayed high mean age, we wondered whether comorbidity development was simply dependent on age. However, age had a low correlation with all of the other features with high importance (Supplementary Fig. S1). In addition, the order of feature importance did not change greatly when the RSF was trained without demographics/anthropometric characteristics. This indicates that comorbidity development was determined not only by age but also other features found important by the RSF (Figs. 3 and 4).

### Automatic clinical assistance through the trained cluster and survival prediction model

**Figure 6:**
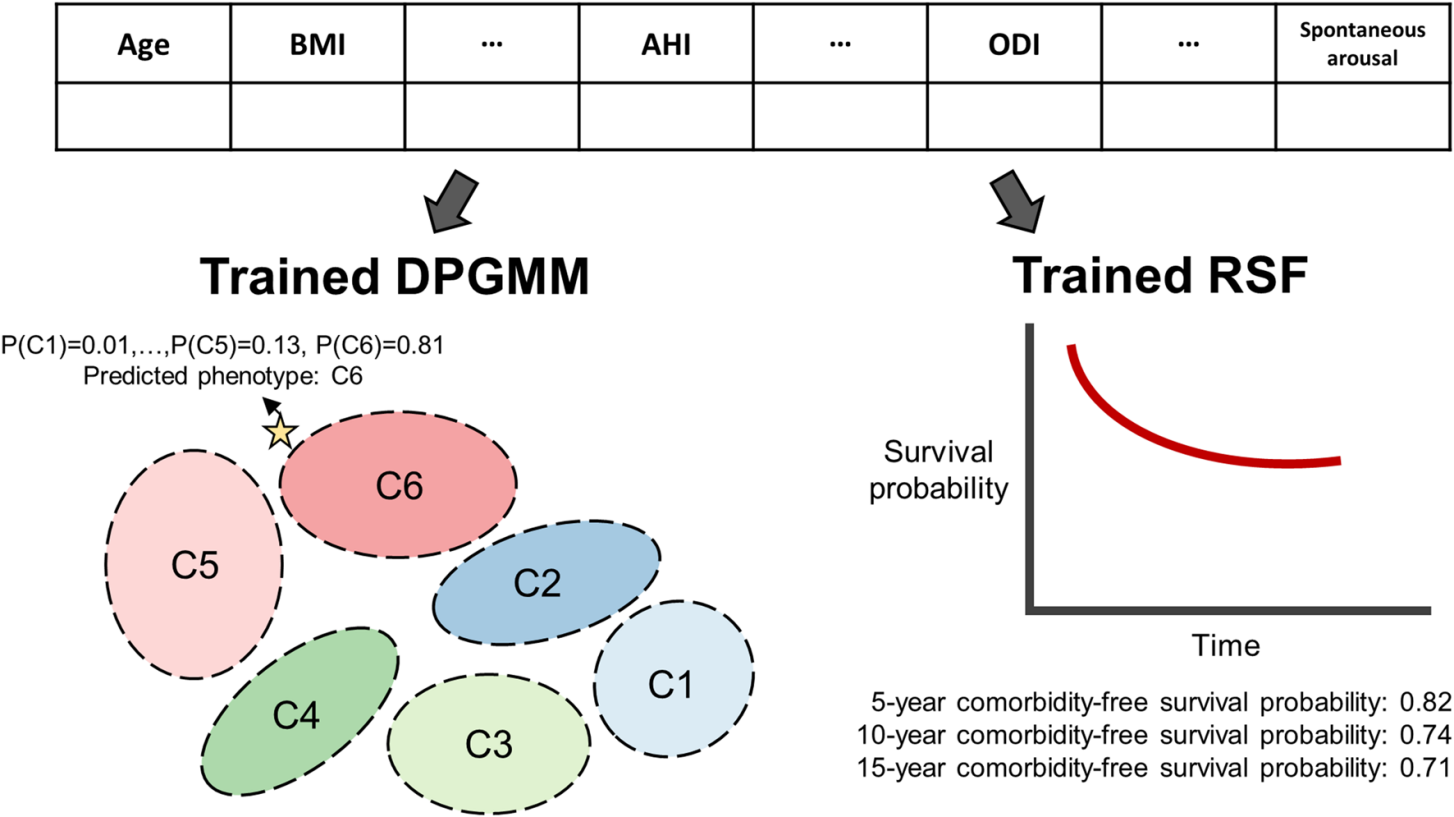
Phenotyping and survival prediction for new patients using the trained DPGMM and RSF. Our computational package calculates the cluster assignment probabilities for new patients based on their 43 PSG features (left) by using the trained DPGMM model in this study (Fig. 1b and Table 1). Furthermore, it predicts their comorbidity risks and the corresponding comorbidity-free survival curve (right) by using the trained RSF in this study (Fig. 1c and Fig. 4).

We have developed a computational package (https://github.com/Mathbiomed/OSA-phenotyping) with the proposed trained models for automatic clinical assistance in OSA patient diagnosis. Based on the PSG data of a patient, the package predicts the phenotype of the patient along with the assignment probabilities for all six clusters (Fig. 6 left). It also predicts the comorbidity-free survival curve of the patient along with the 5-year, 10-year, and 15-year comorbidity-free survival rates. See Supplementary Note S1 for the step-by-step manual for the computational package.

## Discussion

In this study, we discovered new clusters of OSA patients based on all PSG data by applying the DPGMM, which does not require the predetermination of the number of clusters. The identified patient clusters show a statistically significant difference in the prevalence of OSA-associated cardio-neuro-metabolic diseases unlike the conventional AHI-based phenotypes (Fig. 3). Importantly, two clusters had relatively low AHI but high cardio-neuro-metabolic comorbidity risks (Fig. 3). This highlights the importance of considering multimetric PSG data to understand the relationship between OSA and its comorbidities and provides further evidence that single AHI is insufficient for disease diagnosis. Furthermore, in order to describe the difference in the comorbidity prevalence of the discovered clusters based on relevant PSG features (Fig. 5), we used the RSF to identify features with high importance in predicting comorbidity outcomes (Fig. 4).

The clustering algorithms used in previous OSA phenotyping studies^15–18^ required the number of clusters to be manually and potentially subjectively determined. On the other hand, we used DPGMM to cluster OSA patients such that the number of clusters can be inferred from the observed data instead of predetermining it. However, the number of clusters learned from data may change depending on the concentration parameter (a larger concentration parameter more likely yields a higher number of clusters). Although the clustering results were robust to changes in the concentration parameter in our study (Supplementary Table S3), there may be situations where the clustering results may not be as robust. In such cases, the concentration parameter may also be inferred from data by placing a hyperprior on the concentration parameter^41^.

Through cluster analysis using the DPGMM, we found patient clusters with similar mean AHI that show different comorbidity outcomes depending on their PSG features (Figs. 3 and 5). Specifically, a cluster with younger age, lower waist-to-hip ratio, and longer N3 sleep displayed a low prevalence of comorbidities even though it had a moderate degree of mean AHI (C4, mean AHI = 16.6). On the other hand, a cluster with a mild degree of AHI (C2, mean AHI = 8.0) and a cluster with a moderate degree of AHI (C3, mean AHI = 16.1) that displayed opposing characteristics to those of C4 were highly comorbid (Figs. 3b and 5). Importantly, we can use the DPGMM and RSF constructed in this study to identify the phenotypes and predict the comorbidity risks of new patients with our computational package (Fig. 6, Supplementary Fig. S2, and Supplementary Note S1). This provides automatic clinical assistance for OSA patients in various aspects. For example, if the new patients have high probabilities of falling into either C2, C3, C5, or C6 (Fig. 3), clinicians can more closely monitor their risks of cardio-neuro-metabolic diseases regardless of their AHI. For patients with assignment probabilities that are similarly high for multiple clusters, clinicians can observe the patients for characteristics of both phenotypes as well. These are advantages that the probabilistic nature of the DPGMM has over other clustering models that do not require a predetermined number of clusters, such as DBSCAN. Although the monitoring may cause unexpected healthcare costs, it can help prevent cardio-neuro-metabolic diseases via diagnosing and treating the risk factor, OSA, at the appropriate time. Furthermore, the phenotyping can be used for OSA treatment prescriptions although further validation study is necessary in the future. For instance, as patients with AHI ≥ 15 are strongly recommended with positive airway pressure (PAP) therapy^42^, patients of C3 (mean AHI = 16.1) and C4 (mean AHI = 16.6) equally need to get the PAP therapy. However, our work suggests that the PAP therapy appears to be more recommended for C3 than C4 due to a higher risk of cardio-neuro-metabolic diseases compared to C4. Furthermore, our finding suggests that for patients of C2 (mean AHI = 8.0), despite the low AHI level, PAP therapy as well as lifestyle modification might be needed as they have a high risk of the associated diseases. Taken together, incorporation of the cluster analysis and RSF models into the PSG system allows automatic clinical assistance for diagnosis, risk assessment, and treatment of OSA and its associated diseases^43,44^.

Although each identified cluster exhibits multiple key features, not all of these features are always related to the cluster’s comorbidity prevalence. However, previous multimetric cluster-based phenotyping studies for various diseases have resorted to clinical intuition to explain cluster features^15,17,24,25,28^. Thus, we used the nonparametric and nonlinear survival prediction model RSF to investigate the relevance of the PSG features to comorbidity prevalence. Unexpectedly, periodic limb movement (PLM) had extremely low importance according to the RSF (Fig. 4) although PLM was noticeable as a key cluster feature of a high-risk cluster (C3, mean PLM of 39.7) consistent with a previous clustering study^17^. Even though PLM has been modestly associated with increased risks of cardiovascular diseases^10,45^, the high correlation between PLM and other known risk factors such as age^46,47^ raises a question regarding its independent role in comorbidity prevalence. Indeed, the distinguishing features of C3 include not only high PLM but also high age (Fig. 5c), which, unlike PLM, was a highly important feature identified by RSF (Fig. 4). This demonstrates the value of performing a combination of unsupervised and supervised analyses to identify the highly-confident critical features of OSA patients leading to increased risks of associated comorbidities.

Indeed, the key cluster features of the comorbid clusters identified by the RSF to be highly related to cardio-neuro-metabolic disease outcomes were consistent with previous cohort studies that investigated the relationship between various PSG features and the considered diseases. According to the RSF (Fig. 4), the proportion of N3 sleep, REM latency, and Pittsburgh sleep quality index were important features predicting comorbidity development, explaining why a cluster with poor sleep had high comorbidity prevalence despite low mean AHI (Table 1 and Fig. 3). This is consistent with previous studies showing that decreased N3 sleep^48^ and poor sleep quality^49,50^ are associated with increased comorbidity prevalence. Average oxygen saturation, sleep time spent under 90% oxygen saturation, oxygen desaturation index, and lowest oxygen saturation were also features that contribute to increased comorbidity risks as highlighted by the RSF (Figs. 4 and 5), explaining the low comorbidity-free survival rates shown by C5 and C6. This is consistent with previous studies that have shown oxidative stress to be the possible underlying mechanism for OSA triggering comorbidities^51^ and oxygen desaturation to be an independent risk factor for OSA comorbidities^11,38,52,53^. These further support using the identified important features along with the conventional AHI to improve the diagnosis and treatment of OSA. In addition to our model-based analyses, it would be interesting in future work to combine these important features into a single score diagnosis framework for a quick and intuitive representation of the patients’ OSA severity.

The study has several limitations that need consideration. We only considered numeric features and therefore ignored well-known phenotypic features that are not numeric, such as gender, ethnicity, and dentofacial characteristics^54–57^. Although we did not explicitly include gender as a feature, we expect the effects of gender to be implicitly considered since the PSG characteristics differ significantly between genders (Supplementary Table S9). In addition, despite the relatively large number of patients included in the study, we could not investigate the effects of ethnicity and dentofacial characteristics due to lack of data, since the patients were mostly Korean and from the same hospital. It would be important future work to discover multivariate phenotypes through mixed-type data clustering with non-numeric phenotypic features included and to validate the newly identified OSA phenotypes with data from a second site such as the Observational Health Data Science and Informatics (OHDSI) network^58^. For this, we have worked on making the PSG data available via common data model (CDM). To the best of our knowledge, there are no other sites that have made PSG data available via CDM yet. Smoking and drinking history were not considered because the data were only available for about half of the patients. Furthermore, this is a retrospective study, and evaluation of the comorbidity status of all patients was solely based on the diagnosis code from the electronic medical records system of the hospital. This study also does not include the effects of treatment with positive airway pressure therapy because the prescription rate of this treatment was very low as it was not covered by the national health insurance in Korea until 2018. Because the compliance rates of the treatment were low as well (subjective compliance of 34.0% and objective compliance of 20.7%)^59^, we presume the effect of treatment on our analyses to be minimal. The effects of any other interventions during the follow-up were also not considered due to lack of data. With respect to the survival prediction model, we calculated feature importance by comparing the out-of-bag prediction accuracy between actual data and randomly permuted data, as this method is known to be efficient and reliable. However, the feature importance may change depending on the method used to calculate the importance. In addition, our RSF model only had modest predictive power. This decreases the reliability of the feature importance calculated by the RSF (Fig. 4), and thus the key features selected based on the importance might not be the best choice to explain the phenotypes discovered from the cluster analysis (Fig. 5); however, it is still better than the subjective choice of cluster features^17,18,39,40^.

In conclusion, we propose a new multimetric phenotyping framework using the DPGMM and RSF for a better understanding of the pathophysiology of complex diseases with minimized subjective decisions. We applied the framework to data of OSA patients, identifying six new clusters that display comorbidity prevalence unexplainable by the conventional sole AHI. This shows that PSG features should be incorporated in the diagnosis standards for OSA along with the AHI. The cluster model and survival prediction model from this study can be used to phenotype new patients by using their PSG data as inputs to our computational package. Such a diagnosis framework combining unsupervised and supervised models can be applied for the diagnosis and personalized treatment of other major complex and heterogeneous diseases such as sepsis, Parkinson’s disease, and diabetes. The use of this phenotyping framework may lead to the discovery of new phenotypes of these diseases with a focus on any clinical outcome of interest. The phenotyping results can have more practical value when integrated into an electronic medical records system for automatic clinical assistance.

## Methods

### Study subjects

**Figure 7:**
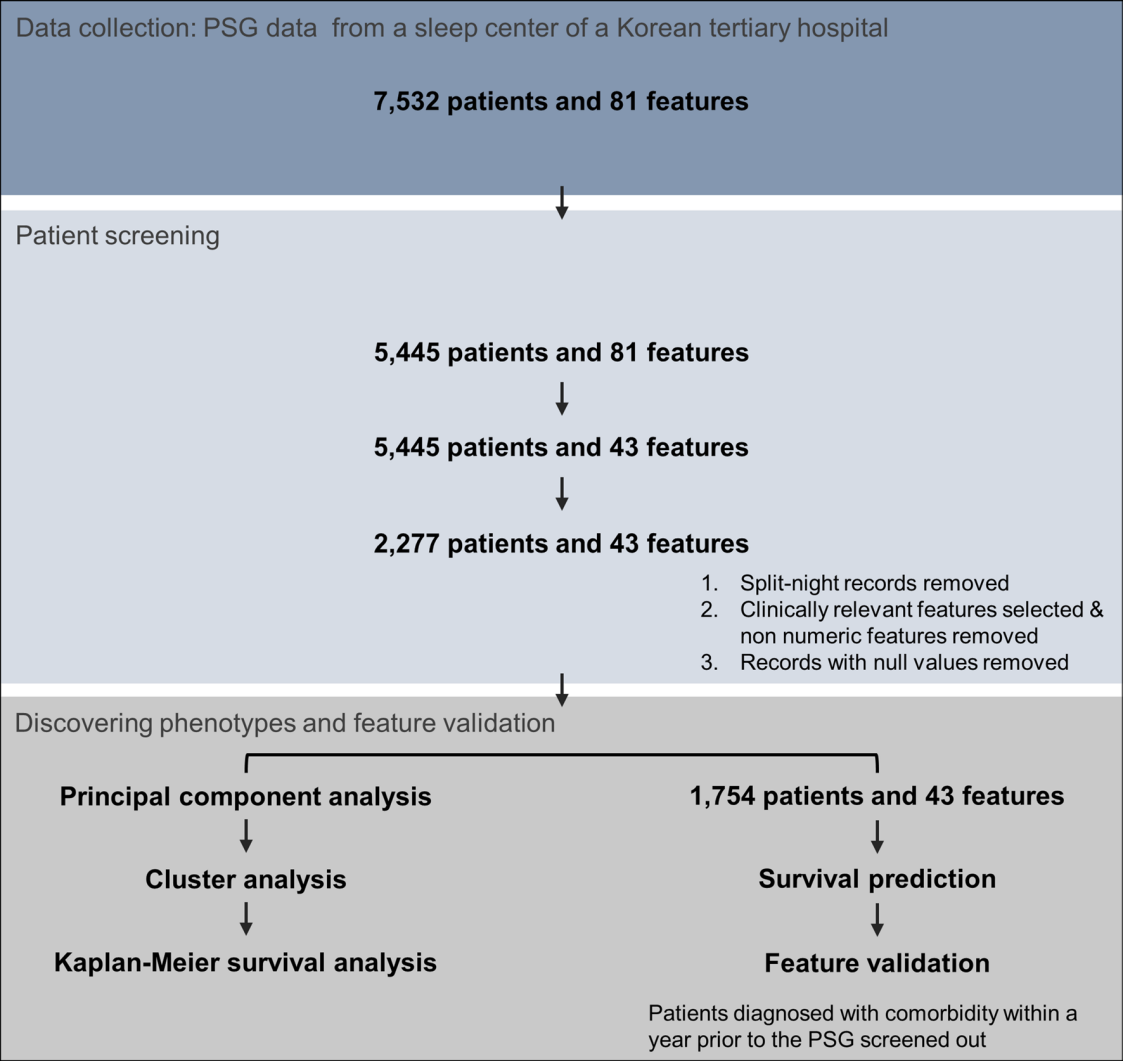
The processing flow of the patient PSG data.

A retrospective study of patients who had undergone the PSG at the sleep center of a tertiary hospital was conducted (Fig. 7). Patients who underwent their first PSG test from 2004 to 2017 were extracted from the Clinical Data Warehouse of hospital electronic medical record system, Bestcare (Ezcaretech, Seoul, Korea) (n = 7532). PSG scores including sleep architecture, respiratory events, respiratory event durations, oxygen saturation information, snoring statistics, limb movement statistics, and arousal statistics, along with demographic/anthropometric characteristics and sleep questionnaire scores of these patients were extracted as the input features for the study. Only patients that took the full-night PSG were included in the study (n = 5445), and patients with any missing values for the variables considered in the study were excluded. As a result, 2277 patients were included in the cluster analysis. For all survival analyses, only the patients who were not diagnosed with the comorbidities considered in the study within a year prior to the PSG were included, resulting in 1754 patients.

The diagnosis information of the 2277 patients based on the International Classification of Disease-10 codes was also extracted from the Clinical Data Warehouse of Bestcare. We included hypertension, dyslipidemia, type 2 diabetes mellitus, ischemic heart disease, atrial fibrillation and flutter, cardiomyopathy, congestive heart failure, aortic aneurysm, and stroke as comorbidities in this study (See Supplementary Table S10 for the International Classification of Disease-10 codes used). The comorbidities were used as a combined label, where an event was considered observed if any one of the comorbidities in consideration was observed, resulting in 249 patients with an event observed. The time between the PSG test and the incidence of that disease was taken as the time-to-event.

### Dimension reduction for cluster analysis

The PSG data is very high dimensional with some highly correlated features. Therefore, we extracted a smaller set of new uncorrelated variables from the original PSG data and used it for the cluster analysis (Fig. 7). For this, we used principal component analysis, which is a dimension reduction technique that linearly transforms a number of possibly correlated features into a small number of uncorrelated variables called principal components. From the initial 81 features, 21 redundant features were removed if the same information could be obtained from another feature (See Supplementary Table S11 for the excluded features). For example, total time in bed was removed as the same information can be obtained through total sleep time and sleep efficiency, and only one feature for the AHI of each sleep position was included. In addition, we removed gender as the algorithms can only handle numeric input. However, the effects of gender are implicitly considered since the PSG characteristics differ significantly between genders (Supplementary Table S9). Furthermore, 16 features with missing values over 10% were also removed as including them reduces the number of valid patient samples drastically. As a result, 43 features were used in the analysis (Table 1 and Supplementary Table S12). While we included most of the features included in previous OSA phenotyping studies^16,17^, it would be an interesting future work to perform phenotyping after adding the excluded features. The selected features were then standardized and decomposed using principal component analysis. Overall, eight principal components explaining up to 70% of the total data variance were used as the input features for the cluster analysis.

### Cluster modeling

The DPGMM^60^ was used to cluster the patients, where each cluster was identified as a distinct phenotype. The DPGMM is a Bayesian nonparametric clustering model that is an extension of the Gaussian mixture model using the Dirichlet process prior^61^ on the mixing proportions. While clustering methods previously used for PSG-based phenotyping such as K-Means clustering^62^ require the number of clusters to be set in advance, the DPGMM infers the number of clusters that best fits the training dataset within a Bayesian statistical framework. The concentration parameter, which may affect the number of clusters created by the algorithm, was chosen as 0.01; the clustering results were in general robust to the changes in the concentration parameter and initializations (Supplementary Table S3 and Supplementary Table S4). Comorbidity outcomes of the clusters were analyzed using survival analysis; Kaplan–Meier curves^63^ were constructed with the combined comorbidity label set as the event of interest.

### Feature identification

The RSF^64^ was used to find key PSG features highly related to comorbidity prevalence, and the clusters identified in the cluster analysis were distinguished based on the features found important by the RSF. The RSF is a nonlinear and nonparametric survival prediction model based on the random forest, where multiple decision trees are grown through bootstrap aggregation and random selection of input variables. The RSF differs from the random forest in that it handles right-censored data: data in which an event may not have been observed. In the RSF, individual trees are grown to calculate the cumulative hazard function for the input sample and the final output is generated by averaging the individual cumulative hazard functions. Moreover, feature importance can be calculated to interpret the predictions made by the model through the difference between the out-of-bag prediction accuracy of the model trained on true data and the model trained on randomly permuted data for the feature. The RSF was preferred over the traditional Cox proportional hazard model to better handle the high dimensional PSG data^64–66^. For the model parameters, the number of input features randomly chosen for each tree was set as 10 and the number of trees to grow was set as 2000. Model performance was measured through fivefold cross-validation. Because the RSF output is in terms of the cumulative hazard function, it is difficult to visualize the comorbidity risks of the patients with respect to time. Therefore, the cumulative hazard function was transformed into a survival curve through the relationship $S\left(t\right)=\mathrm{exp}\left(-\Lambda \left(t\right)\right)$*,*where S(t) and Λ(t) are the survival function and the cumulative hazard function, respectively.

The cluster analysis and visualizations were conducted using open-source libraries of Python 3.7 (scikit-learn 0.20^67^, pandas 0.23^68^, lifelines 0.15^69^, matplotlib 3.0.2^70^). The survival prediction analysis for feature validation was conducted using packages of R.3.4.2 (randomforestSRC 2.7.0^64^, pec^71^).

### Ethics statement

All methods were conducted in accordance with relevant guidelines (Declaration of Helsinki) and regulations. The authors of this manuscript conducted a retrospective review of patient data who had undergone polysomnography. The present study had been approved by the Institutional Review Board of Seoul National University Bundang Hospital (IRB No.B-1804/465-104)) and the waiver of consent had been approved by the IRB since this study involved only a retrospective review of anonymous patient data.

## Supplementary Information

Supplementary Information.

## Data Availability

The data cannot be publicized for legal reasons. The computational code for predicting the phenotypes and comorbidity risks of new patients using their PSG data, along with the trained models and a sample test dataset of a patient in csv format, are provided at https://github.com/Mathbiomed/OSA-phenotyping.

## References

[1] Lee W, Nagubadi S, Kryger MH, Mokhlesi B (2008). Epidemiology of obstructive sleep apnea: A population-based perspective. Expert Rev. Resp. Med.

[2] Shahar E, Whitney CW, Redline S, Lee ET, Newman AB, Javier Nieto F, O'Connor GT, Boland LL, Schwartz JE, Samet JM (2001). Sleep-disordered breathing and cardiovascular disease: Cross-sectional results of the sleep heart health study. Am. J. Respir. Crit. Care Med..

[3] Peker Y, Hedner J, Norum J, Kraiczi H, Carlson J (2002). Increased incidence of cardiovascular disease in middle-aged men with obstructive sleep apnea: A 7-year follow-up. Am. J. Respir. Crit. Care Med..

[4] Yaggi HK (2005). Obstructive sleep apnea as a risk factor for stroke and death. N. Engl. J. Med..

[5] Bradley TD, Floras JS (2009). Obstructive sleep apnoea and its cardiovascular consequences. Lancet.

[6] Marshall NS (2009). Is sleep apnea an independent risk factor for prevalent and incident diabetes in the Busselton health study?. J. Clin. Sleep Med..

[7] Kendzerska T, Gershon AS, Hawker G, Tomlinson G, Leung RS (2014). Obstructive sleep apnea and incident diabetes a historical cohort study. Am. J. Respir. Crit. Care Med..

[8] Sateia MJ (2014). International classification of sleep disorders-third edition highlights and modifications. Chest.

[9] Gastaut H, Tassinari CA, Duron B (1966). Polygraphic study of the episodic diurnal and nocturnal (hypnic and respiratory) manifestations of the pickwick syndrome. Brain Res..

[10] Kendzerska T (2014). Untreated obstructive sleep apnea and the risk for serious long-term adverse outcomes: A systematic review. Sleep Med. Rev..

[11] Hoffman AR (2016). Sleep disordered breathing and risk of stroke in older community-dwelling men. Sleep.

[12] Kulkas A, Tiihonen P, Julkunen P, Mervaala E, Töyräs J (2013). Novel parameters indicate significant differences in severity of obstructive sleep apnea with patients having similar apnea-hypopnea index. Med. Biol. Eng. Comput..

[13] Vavougios GD, Natsios G, Pastaka C, Zarogiannis SG, Gourgoulianis KI (2016). Phenotypes of comorbidity in OSAS patients: Combining categorical principal component analysis with cluster analysis. J. Sleep Res..

[14] Zinchuk AV, Gentry MJ, Concato J, Yaggi HK (2017). Phenotypes in obstructive sleep apnea: A definition, examples and evolution of approaches. Sleep Med. Rev..

[15] Lacedonia D (2016). Characterization of obstructive sleep apnea–hypopnea syndrome (OSA) population by means of cluster analysis. J. Sleep Res..

[16] Joosten SA (2012). Phenotypes of patients with mild to moderate obstructive sleep apnoea as confirmed by cluster analysis. Respirology.

[17] Zinchuk AV (2017). Polysomnographic phenotypes and their cardiovascular implications in obstructive sleep apnoea. Thorax.

[18] Ye L (2014). The different clinical faces of obstructive sleep apnoea: A cluster analysis. Eur. Respir. J..

[19] Haldar P (2008). Cluster analysis and clinical asthma phenotypes. Am. J. Respir. Crit. Care Med..

[20] Siroux V (2011). Identifying adult asthma phenotypes using a clustering approach. Eur. Respir. J..

[21] Wu W (2014). Unsupervised phenotyping of Severe Asthma Research Program participants using expanded lung data. J. Allergy Clin. Immunol..

[22] Schatz M (2014). Phenotypes determined by cluster analysis in severe or difficult-to-treat asthma. J. Allergy Clin. Immunol..

[23] Burgel PR (2010). Clinical COPD phenotypes: A novel approach using principal component and cluster analyses. Eur. Respir. J..

[24] Garcia-Aymerich J (2011). Identification and prospective validation of clinically relevant chronic obstructive pulmonary disease (COPD) subtypes. Thorax.

[25] Ahmad T (2014). Clinical implications of chronic heart failure phenotypes defined by cluster analysis. J. Am. Coll. Cardiol..

[26] Seymour CW (2019). Derivation, validation, and potential treatment implications of novel clinical phenotypes for sepsis. J. Am. Med. Assoc..

[27] Fereshtehnejad SM (2015). New clinical subtypes of Parkinson disease and their longitudinal progression a prospective cohort comparison with other phenotypes. JAMA Neurol..

[28] Ahlqvist E (2018). Novel subgroups of adult-onset diabetes and their association with outcomes: A data-driven cluster analysis of six variables. Lancet Diabetes Endocrinol..

[29] Udler MS (2018). Clustering of type 2 diabetes genetic loci by multi-trait associations identifies disease mechanisms and subtypes. PLoS Med..

[30] Kadane JB, Lazar NA (2004). Methods and criteria for model selection. J. Am. Stat. Assoc..

[31] Jain AK (2010). Data clustering: 50 years beyond K-means. Pattern Recognit. Lett..

[32] Ding J, Tarokh V, Yang Y (2018). Model selection techniques: An overview. IEEE Signal Process. Mag..

[33] Yu, G., Huang, R. & Wang, Z. Document clustering via dirichlet process mixture model with feature selection. In *Proc. ACM SIGKDD Int. Conf. Knowl. Discov. Data Min.* 763–771 (2010) 10.1145/1835804.1835901.

[34] Lim DC, Sutherland K, Cistulli PA, Pack AI (2017). P4 medicine approach to obstructive sleep apnoea. Respirology.

[35] White N, Johnson H, Silburn P, Mengersen K (2012). Dirichlet process mixture models for unsupervised clustering of symptoms in Parkinson’s disease. J. Appl. Stat..

[36] Barrera C (2019). Phenotyping tumor infiltrating lymphocytes (PhenoTIL) on H&E tissue images: Predicting recurrence in lung cancer. Proc. SPIE.

[37] Vermunt JK (2011). K-means may perform as well as mixture model clustering but may also be much worse: Comment on Steinley and Brusco (2011). Psychol. Methods.

[38] Kendzerska T, Gershon AS, Hawker G, Leung RS, Tomlinson G (2014). Obstructive sleep apnea and risk of cardiovascular events and all-cause mortality: A decade-long historical cohort study. PLoS Med..

[39] Bailly S (2016). Obstructive sleep apnea: A cluster analysis at time of diagnosis. PLoS ONE.

[40] Butler MP (2018). Apnea-hypopnea event duration predicts mortality in men and women in the Sleep Heart Health Study. Am. J. Respir. Crit. Care Med..

[41] Gershman, S. J. & Blei, D. M. A Tutorial on Bayesian Nonparametric Models. 1–28 (2011) 10.1016/j.jmp.2011.08.004.

[42] Patil SP (2019). Treatment of adult obstructive sleep apnea with positive airway pressure: An American academy of sleep medicine systematic review, meta-analysis, and GRADE assessment. J. Clin. Sleep Med..

[43] Mandel JC, Kreda DA, Mandl KD, Kohane IS, Ramoni RB (2016). SMART on FHIR: A standards-based, interoperable apps platform for electronic health records. J. Am. Med. Informatics Assoc..

[44] Girdea M (2013). PhenoTips: Patient phenotyping software for clinical and research use. Hum. Mutat..

[45] Koo BB, Sillau S, Dean DA, Lutsey PL, Redline S (2015). Periodic limb movements during sleep and prevalent hypertension in the multi-ethnic study of atherosclerosis. Hypertension.

[46] Ancoli-Israel S (1991). Periodic limb movements in sleep in community-dwelling elderly. Sleep.

[47] Scofield H, Roth T, Drake C (2008). Periodic limb movements during sleep: Population prevalence, clinical correlates, and racial differences. Sleep.

[48] Fung MM (2011). Decreased slow wave sleep increases risk of developing hypertension in elderly men. Hypertension.

[49] Hayashino Y (2010). Association between number of comorbid conditions, depression, and sleep quality using the Pittsburgh Sleep Quality Index: Results from a population-based survey. Sleep Med..

[50] Hoevenaar-Blom MP, Spijkerman AMW, Kromhout D, van den Berg JF, Verschuren WMM (2011). Sleep duration and sleep quality in relation to 12-year cardiovascular disease incidence: The MORGEN Study. Sleep.

[51] Lavie L (2003). Obstructive sleep apnoea syndrome—An oxidative stress disorder. Sleep Med. Rev..

[52] Nieto, F. J. *et al.* In a Large Community-Based Study for the Sleep Heart Health Study, Vol. 283, 1829–1837 (2000). 10.1001/jama.283.14.1829 10770144

[53] Tkacova R (2014). Nocturnal intermittent hypoxia predicts prevalent hypertension in the European Sleep Apnoea Database cohort study. Eur. Respir. J..

[54] Ye L, Pien GW, Weaver TE (2009). Gender differences in the clinical manifestation of obstructive sleep apnea. Sleep Med..

[55] Subramanian S (2011). Gender and ethnic differences in prevalence of self-reported insomnia among patients with obstructive sleep apnea. Sleep Breath..

[56] Eckert DJ, White DP, Jordan AS, Malhotra A, Wellman A (2013). Defining phenotypic causes of obstructive sleep apnea: Identification of novel therapeutic targets. Am. J. Respir. Crit. Care Med..

[57] Hui DS (2017). Differences in craniofacial structures and obesity in caucasian and Chinese patients with obstructive sleep apnea. Sleep.

[58] OHDSI—Observational Health Data Sciences and Informatics. https://www.ohdsi.org/.

[59] Choi J-A, Yoon I-Y, Han E-G, Lee S (2011). Subjective and objective CPAP compliance in patients with obstructive sleep apnea syndrome. Sleep Med. Res..

[60] Rasmussen CE (2000). The infinite Gaussian mixture model. Adv. Neural Inf. Process. Syst..

[61] Ferguson TS (1973). A Bayesian analysis of some nonparametric problems. Ann. Stat..

[62] MacQueen, J. Some methods for classification and analysis of multivariate observations. In *Proc. 5th Berkeley Symp. Math. Stat. Probab.* 281–297 (1967) 10.1007/s11665-016-2173-6.

[63] Kaplan EL, Meier P (1958). Nonparametric estimation from incomplete observations. J. Am. Stat. Assoc..

[64] Ishwaran H, Kogalur UB, Blackstone EH, Lauer MS (2008). Random survival forests. Ann Appl. Stat..

[65] Hsich E, Gorodeski EZ, Blackstone EH, Ishwaran H, Lauer MS (2011). Identifying important risk factors for survival in patient with systolic heart failure using random survival forests. Circ. Cardiovasc. Qual. Outcomes.

[66] Simsek B (2017). Which sleep health characteristics predict all-cause mortality in older men? An application of flexible multivariable approaches. Sleep.

[67] Pedregosa F (2011). Scikit-learn: Machine learning in Python. JMLR.

[68] Mckinney, W. & Pydata Development Team. Pandas : Powerful python data analysis toolkit release 0.13.1. *Python Packag.* 1211 (2014).

[69] Davidson-Pilon, C. *et al.* CamDavidsonPilon/lifelines: v0.21.0. (2019) 10.5281/ZENODO.2638135.

[70] Hunter JD (2007). Matplotlib: A 2D graphics environment. Comput. Sci. Eng..

[71] Mogensen UB, Ishwaran H, Gerds TA (2012). Evaluating random forests for survival analysis using prediction error curves. J. Stat. Softw..

