# Supplementary material for: Combined unsupervised-supervised machine learning for phenotyping complex diseases with its application to obstructive sleep apnea
Source: Sci Rep. 2021 Feb 24;11:4457. doi: 10.1038/s41598-021-84003-4 (PMC7904925; doi:10.1038/s41598-021-84003-4)
Supplement: Supplementary file 1 — Supplementary Information. [file 41598_2021_84003_MOESM1_ESM.pdf]

Combined unsupervised-supervised machine learning for phenotyping complex diseases with its application to obstructive sleep apnea

Authors

Eun-Yeol Ma[1†], Jeong-Whun Kim[2†], Youngmin Lee[1], Sung-Woo Cho[2], Heeyoung Kim[1**], Jae Kyoung Kim[3*]

Affiliations

[1] Department of Industrial and Systems Engineering, Korea Advanced Institute of Science and Technology, Daejeon, Republic of Korea, [2] Department of Otorhinolaryngology, Seoul National University Bundang Hospital, Seongnam, Republic of Korea, [3] Department of Mathematical Sciences, Korea Advanced Institute of Science and Technology, Daejeon, Republic of Korea

[†]These authors contributed equally to this work.

[*] Correspondence: jaekkim@kaist.ac.kr

[**] Co-correspondence: heeyoungkim@kaist.ac.kr

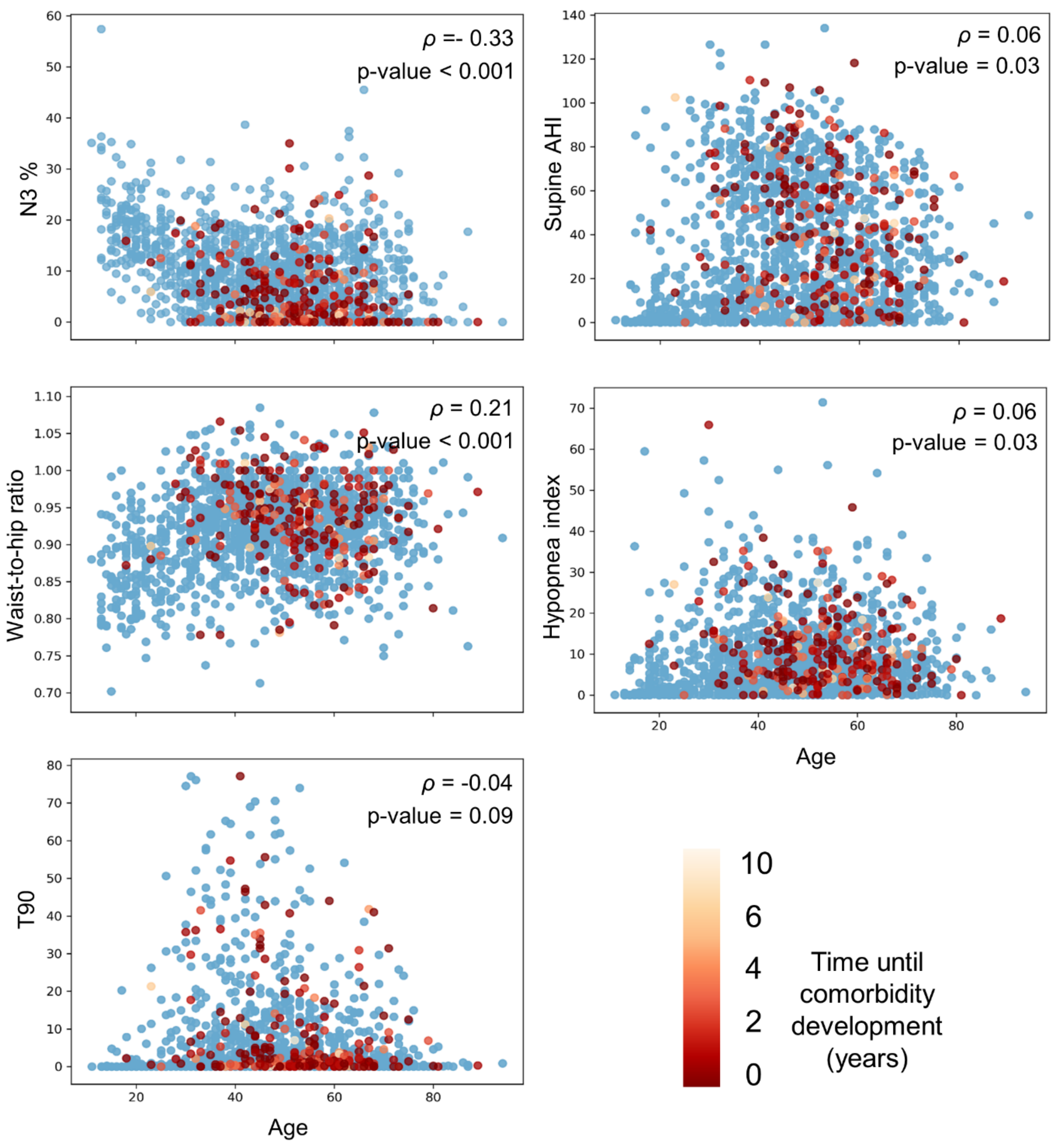

Supplementary Fig. S1 Correlation of selected PSG features with age. The comorbidity development status was color-coded based on the time-to-event. Blue dots represent the patients free of comorbidities during the follow-up period. Age had a low correlation with all of the other features with high importance, and comorbidity development displayed no particular pattern regarding these features.

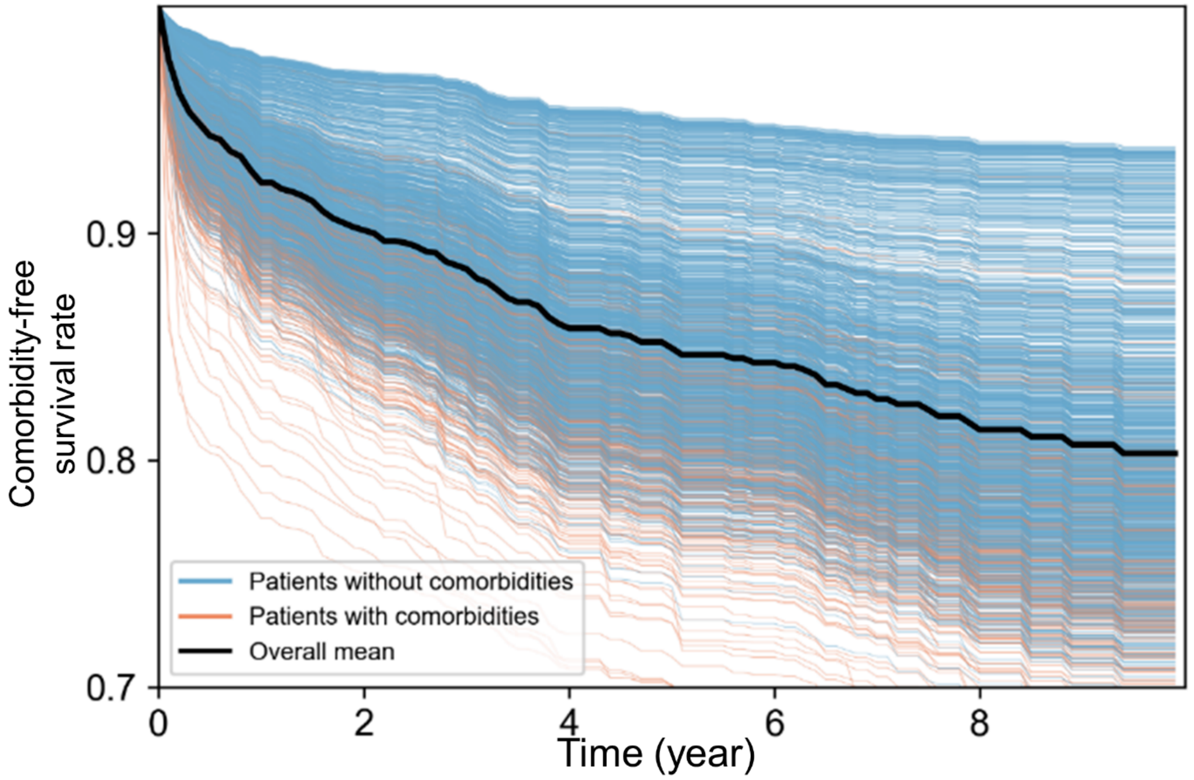

Supplementary Fig. S2 Comorbidity-free survival curve predictions by the RSF. The survival curves were color-coded based on the true event status. The patients with highly predicted comorbidity risks (high cumulative hazard function) according to the RSF had more rapidly falling survival curves in general. RSF, random survival forest.

Supplementary Table S1 AHI-based OSA severity diagnosis standards.

OSA phenotype	AHI range
None	$AHI < 5$
Mild	$5 \leq AHI < 15$
Moderate	$15 \leq AHI < 30$
Severe	$30 \leq AHI$

Supplementary Table S2 Silhouette width and Bayesian information criterion of K-means clustering.

Number of clusters	Silhouette width	Bayesian information criterion
3	0.18	35483.8
4	0.18	35156.5
5	0.18	34866.1
6	0.15	34648.2
7	0.14	34638.5
8	0.14	34615.6
9	0.13	34566.1
10	0.13	34480.0

In general, higher values of silhouette width and lower values of the Bayesian information criterion is preferred. It is difficult to choose the optimal number of clusters by looking at only the selection criteria, as a K-means model with a small number of clusters is preferred when looking at the silhouette width while more clusters are suggested by the Bayesian information criterion.

Supplementary Table S3 Number of clusters discovered with varying concentration parameter

Concentration parameter	0.001	0.005	0.01	0.1	1.0	10
No. of clusters	5	6	6	6	6	6

Supplementary Table S4 Proportion of the number of clusters inferred for 1,000 repetitions of DPGMM experiments.

No. of clusters	5	6	7
Proportion (%)	22.0	64.7	13.3

Supplementary Table S5 Analysis of variance results for pairwise comparison of feature means between clusters of similar mean AHI.

	C1 mean	C2 mean	pairwise p-value	C3 mean	C4 mean	pairwise p-value	C5 mean	C6 mean	pairwise p-value
Age	36.4	54.3	0.0	60.0	45.8	0.0	47.4	52.8	0.0

BMI	23.3	23.7	0.1	25.3	24.6	0.0	27.8	27.3	0.0
Neck circumference	34.9	35.0	0.7	36.9	37.1	0.3	39.4	39.3	0.4
Waist-Hip ratio	0.9	0.9	0.0	0.9	0.9	0.0	1.0	0.9	0.0
PSQI	7.9	9.8	0.0	8.3	7.1	0.0	7.1	8.0	0.0
ESS	10.0	7.6	0.0	8.1	9.6	0.0	9.9	9.9	0.9
Sleep latency	16.1	22.5	0.0	23.4	10.8	0.0	11.8	25.0	0.0
Sleep period time	445.9	444.7	0.7	438.3	446.6	0.0	448.7	422.6	0.0
WASO	32.8	93.6	0.0	85.0	52.0	0.0	62.8	94.9	0.0
Total sleep time	414.1	356.7	0.0	358.2	396.1	0.0	387.5	334.2	0.0
Sleep efficiency	89.4	75.5	0.0	77.0	86.5	0.0	84.0	73.9	0.0
REM latency	109.8	138.7	0.0	139.7	110.7	0.0	126.8	145.8	0.0
N1 (%)	5.9	9.3	0.0	10.9	9.6	0.0	14.2	19.9	0.0
N2 (%)	54.4	46.2	0.0	49.6	51.2	0.0	48.8	43.8	0.0
N3 (%)	12.9	10.0	0.0	6.7	9.9	0.0	7.3	3.9	0.0
REM (%)	19.6	14.9	0.0	14.8	18.0	0.0	16.1	11.6	0.0
AHI	3.5	8.0	0.0	16.1	16.6	0.6	36.0	57.3	0.0
Apnea index	1.0	2.8	0.0	8.0	8.4	0.5	20.9	45.4	0.0
Obstructive apnea	0.7	2.4	0.0	6.8	7.6	0.2	18.2	37.1	0.0
Central apnea	0.2	0.3	0.4	0.4	0.4	0.8	0.9	1.8	0.0
Mixed apnea	0.0	0.2	0.0	0.8	0.5	0.1	2.0	6.5	0.0
Hypopnea index	2.5	5.2	0.0	8.1	8.2	1.0	15.1	11.9	0.0
REM AHI	5.9	10.5	0.0	18.3	20.7	0.1	38.1	46.2	0.0
NREM AHI	2.8	7.5	0.0	15.5	15.3	0.9	35.5	58.9	0.0
Supine AHI	5.1	13.4	0.0	25.9	24.7	0.4	51.3	64.9	0.0
Lateral AHI	1.3	2.7	0.0	10.6	6.2	0.0	25.0	53.0	0.0
Longest apnea duration	19.5	26.6	0.0	35.9	44.8	0.0	46.2	66.0	0.0
Mean apnea duration	13.6	16.7	0.0	19.3	23.3	0.0	22.1	29.4	0.0
Mean hypopnea duration	22.0	24.9	0.0	26.1	29.1	0.0	25.5	27.3	0.0
Mean total AH duration	20.7	23.5	0.0	24.9	27.6	0.0	24.5	30.4	0.0
Average O2 saturation	96.8	96.1	0.0	95.4	96.1	0.0	94.7	92.7	0.0
Lowest O2 saturation	90.2	88.6	0.0	85.5	84.4	0.0	79.5	74.4	0.0
T90 (%)	0.2	0.4	0.0	2.2	1.6	0.1	6.0	20.2	0.0
ODI	2.1	5.0	0.0	12.0	11.4	0.5	29.9	51.3	0.0
Snoring time	9.6	8.8	0.3	24.4	29.4	0.0	39.9	19.6	0.0
Number of snoring episodes	35.3	37.1	0.6	79.4	96.8	0.0	167.3	189.8	0.0
Average snoring duration	0.9	0.7	0.0	1.1	1.4	0.0	1.1	0.4	0.0
Longest snoring duration	6.5	5.3	0.0	12.5	17.5	0.0	19.7	4.9	0.0
Limb movement	5.9	10.9	0.0	56.8	7.2	0.0	10.8	21.2	0.0
PLM	0.9	3.9	0.0	39.7	0.5	0.0	0.9	1.4	0.0
Respiratory arousal	2.1	5.3	0.0	11.2	11.8	0.5	25.7	48.1	0.0

PLM arousal	0.2	0.7	0.0	6.9	0.1	0.0	0.1	0.1	0.1
Spontaneous arousal	6.1	6.2	0.8	4.1	5.1	0.0	3.5	2.0	0.0

Supplementary Table S6 Average follow-up time (year) of DPGMM-created clusters.

	C1	C2	C3	C4	C5	C6
Mean (SD)	6.4 (3.3)	5.2 (3.0)	5.4 (3.3)	6.3 (3.5)	6.0 (3.9)	5.3 (3.5)

Supplementary Table S7 The proportion of patients who smoke/drink (n=941).

	C1	C2	Pairwise p-value	C3	C4	Pairwise p-value	C5	C6	Pairwise p-value
Smoking patients (%)	24.4 (22/90)	26.9 (35/130)	0.68	41.3 (59/143)	40.0 (52/130)	0.83	47.3 (124/262)	39.8 (74/186)	0.11
Drinking patients (%)	31.1 (28/90)	45.0 (58/130)	0.04	46.9 (67/143)	49.2 (64/130)	0.69	66.8 (175/262)	57.4 (105/183)	0.04

Supplementary Table S8 The relative importance of the features for comorbidity risk prediction by the RSF.

Feature	Relative importance
Age	1.00
Proportion of N3 sleep	0.40
Waist-hip ratio	0.24
Proportion of sleep spent under 90% oxygen saturation	0.20
Average oxygen saturation	0.16
Oxygen desaturation event index	0.16
Lowest oxygen saturation	0.12
Supine apnea-hypopnea index	0.11
Hypopnea index	0.10
Number of snoring episodes	0.09
Lateral apnea-hypopnea index	0.08
REM latency	0.05
Mixed apnea	0.05
REM apnea-hypopnea index	0.05
Apnea-hypopnea index	0.04
Pittsburgh sleep quality index	0.04

NREM apnea-hypopnea index	0.03
Central apnea	0.03
Snoring %	0.03
Respiratory arousal	0.03
Obstructive apnea	0.02
Apnea index	0.02
Neck circumference	0.02
Proportion of N1 sleep	0.01
Total sleep time	0.01
Body mass index	0.01
Mean total apnea-hypopnea duration	0.01
Proportion of REM sleep	0.01
Longest apnea duration	0.01
Spontaneous arousal	0.01
Mean hypopnea duration	0.00
Wake time after sleep onset	0.00
Mean apnea duration	0.00
Limb movement	0.00
Periodic limb movements arousal	0.00
Sleep period time	0.00
Sleep efficiency	0.00
Proportion of N2 sleep	0.00
Average snoring duration	0.00
Periodic limb movements	0.00
Epworth sleepiness scale	-0.01
Sleep latency	-0.01
Longest snoring duration	-0.01

Supplementary Table S9 Analysis of variance of for comparison of feature means between genders.

	Male mean	Female mean	P-value
n	1730	547	
Age	48.1	54.5	<0.001
BMI	26.1	24.5	<0.001
Neck circumference	38.8	33.3	<0.001
Waist-Hip ratio	0.9	0.9	<0.001
PSQI	7.4	9.4	<0.001

ESS	9.7	7.9	<0.001
Sleep latency	15.2	25.0	<0.001
Sleep period time	442.5	438.4	0.09
WASO	70.6	70.9	0.91
Total sleep time	375.0	371.9	0.29
Sleep efficiency	81.5	79.6	<0.001
REM latency	125.0	141.9	<0.001
N1 (%)	13.7	8.0	<0.001
N2 (%)	48.4	49.8	0.01
N3 (%)	7.0	11.56	<0.001
REM (%)	15.8	15.4	0.29
AHI	29.5	14.6	<0.001
Apnea index	19.3	6.8	<0.001
Obstructive apnea	16.2	6.2	<0.001
Central apnea	0.86	0..28	<0.001
Mixed apnea	2.3	0.32	<0.001
Hypopnea index	10.2	7.7	<0.001
REM AHI	28.4	19.2	<0.001
NREM AHI	29.5	13.3	<0.001
Supine AHI	40.1	18.5	<0.001
Lateral AHI	22.0	8.6	<0.001
Longest apnea duration	46.9	26.3	<0.001
Mean apnea duration	23.3	15.2	<0.001
Mean hypopnea duration	26.9	23.1	<0.001
Mean total AH duration	26.6	22.0	<0.001
Average O2 saturation	94.9	95.7	<0.001
Lowest O2 saturation	81.7	86.4	<0.001
T90 (%)	6.7	2.4	<0.001
ODI	24.3	11.6	<0.001
Snoring time	26.8	17.7	<0.001
Number of snoring episodes	127.9	67.0	<0.001
Average snoring duration	1.0	10	0.25
Longest snoring duration	13.0	9.8	<0.001
Limb movement	18.1	17.7	0.66
PLM	6.2	9.1	0.001
Respiratory arousal	22.9	9.0	<0.001
PLM arousal	1.0	1.9	<0.001
Spontaneous arousal	4.2	4.6	0.02

Supplementary Table S10 ICD-10 codes for comorbidities considered.

Disease	ICD-10 Code
Hypertension	I10-13
Dyslipidemia	E78
Diabetes Mellitus Type 2	E11-E14
Ischemic Heart Disease	I20-I25
Atrial Fibrillation and Flutter	I48
Congestive Heart Failure	I50
Aortic Aneurysm	I71
Cardiomyopathy	I42-I43
Stroke	G45-G46, I60-I63, I67-I69

Supplementary Table S11 Features excluded from the study.

Reason for exclusion	Number of features removed	Excluded features
Redundancy with other features	21	Height<br>Weight<br>Waist circumference<br>Hip circumference<br>Total time in bed<br>Sleep stage & positional respiratory events (min, proportion) (10 features)<br>Hypopnea index with/without oxygen desaturation<br>Number of awakenings<br>Number of movement time epochs<br>Snoring time (min)<br>Limb movement with arousal
Missing value > 10%	16	Mortality-related information (3 features)<br>Beck Depression Inventory<br>Friedman stage information (4 features)<br>Nocturia information (2 features)<br>Prone AHI information (3 features)<br>Waking oxygen saturation<br>Time spent with oxygen saturation below 60%/70%/80%
Categorical	1	Gender

Supplementary Table S12 Features used in the study and full cohort mean (SD) (n=2277).

Feature category	Variable	Mean (SD)
Demographic and anthropometric characteristics	Age	49.6 (15.3)
	Body mass index (BMI)	25.7 (3.8)
	Neck circumference	37.5 (3.5)

	Waist-Hip ratio	0.9 (0.1)
Questionnaires, sleep quality and sleep architecture	Pittsburgh sleep quality index (PSQI)	7.9 (4.2)
	Epworth sleepiness scale (ESS)	9.3 (5.1)
	Sleep latency (min)	17.6 (24.4)
	Sleep period time (min)	441.5 (49.7)
	Wake time after sleep onset (WASO) (min)	70.7 (48.1)
	Total sleep time (min)	374.2 (60.6)
	Sleep efficiency (%)	81.1 (11.7)
	REM latency from sleep onset (min)	129.1 (76.1)
	Time of stage N1 NREM sleep (N1) (%)	12.3 (8.2)
	Time of stage N2 NREM sleep (N2) (%)	48.7 (11.5)
	Time of stage N3 NREM sleep (N3) (%)	8.1 (7.5)
	Time of REM sleep (%)	15.7 (6.7)
Respiratory events	Apnea-hypopnea index (AHI) (/hr)	25.9 (23.9)
	Apnea index (/hr)	16.3 (20.5)
	Obstructive apnea (/hr)	13.8 (18.0)
	Central apnea (/hr)	0.7 (2.9)
	Mixed apnea (/hr)	1.9 (5.2)
	Hypopnea index (/hr)	9.6 (0.3)
	REM AHI (/hr)	27.2 (23.8)
	NREM AHI (/hr)	25.6 (25.0)
	Supine AHI (/hr)	34.9 (29.0)
	Lateral AHI (/hr)	18.8 (35.0)
Durations	Longest apnea duration (sec)	42.0 (25.9)
	Mean apnea duration (sec)	21.4 (9.4)
	Mean hypopnea duration (sec)	26.0 (8.7)
	Mean total apnea-hypopnea duration (sec)	25.5 (7.8)
SpO2	Average oxygen saturation (%)	95.1 (2.3)
	Lowest oxygen saturation (%)	82.8 (8.5)
	Time spent below 90% oxygen saturation (T90) (%)	5.7 (11.6)
	Oxygen desaturation index (ODI) (/hr)	21.2 (22.5)
Snoring	Snoring time (%)	24.6 (20.6)
	Number of snoring episodes	113.3 (101.2)

	Average snoring episode duration (min)	1.0 (0.9)
	Longest snoring episode duration (min)	12.2 (12.7)
PLM index	Limb movement (/hr)	18.03 (23.3)
	Periodic limb movement (PLM) (/hr)	6.91 (17.6)
Arousal index	Respiratory arousal (/hr)	19.56 (20.6)
	PLM arousal (/hr)	1.17 (4.4)
	Spontaneous arousal (/hr)	4.29 (3.8)

Supplementary Note S1 Step-by-step manual on the computational package usage (Fig. 6).

Step 1. *Download 'predict_DPGMM.py', 'predict_RSF.R', 'cluster_idx.pkl', 'DPGMM_model.pkl', 'PCA_weights.pkl', 'population_mean.csv', 'population_std.csv', 'random_testset.csv', 'test_input.csv', 'RSF_model.rda' files from https://github.com/Mathbiomed/OSA-phenotyping and place them in the same directory..*
Step 2. *Fill in the PSG data of the patient as the 1st row (index 0) of the 'test_input.csv' file. The data must match the column heading of the csv file, which is ordered as it was in our study. The units for each feature must match the units listed in the csv file.*
Step 3. *Run 'predict_DPGMM.py' with Python3 to obtain the predicted phenotype and cluster assignment probabilities*. The outputs, which are the cluster assignment probabilities and the final predicted phenotype, will be printed out. *Pickle, Numpy, Pandas, and Scikit-learn* libraries must be installed before running. You may want to change line 19 to a comment (add # in front) and uncomment (delete # in front) line 20 of 'predict_DPGMM.py' to test how the code runs with a sample test dataset of a patient.
Step 4. *Run 'predict_RSF.rda' with R to obtain the predicted comorbidity-free survival probabilities and the corresponding survival curve*. The outputs, which are the 5-year, 10-year, and 15-year comorbidity-free survival probabilities, will be printed out. The corresponding survival curve will appear as a plot. The *randomForestSRC* and *pec* libraries must be installed before running 'predict_RSF.rda'. You may want to change line 6 to a comment (add # in front) and uncomment line 7 of 'predict_RSF.rda' to test how the code runs with a sample test dataset of a patient."
